# Smoking Is Associated with an Increased Risk of Dementia: A Meta-Analysis of Prospective Cohort Studies with Investigation of Potential Effect Modifiers

**DOI:** 10.1371/journal.pone.0118333

**Published:** 2015-03-12

**Authors:** Guochao Zhong, Yi Wang, Yong Zhang, Jeff Jianfei Guo, Yong Zhao

**Affiliations:** 1 The Second College of Clinical Medicine, Chongqing Medical University, Chongqing, China; 2 School of Public Health and Management, Chongqing Medical University, Chongqing, China; 3 Division of Pharmacy Practice and Administrative Sciences, College of Pharmacy, University of Cincinnati, Cincinnati, OH, 45221, United States of America; University of Hertfordshire, UNITED KINGDOM

## Abstract

**Background:**

Previous studies showed inconsistent results on the association of smoking with all-cause dementia and vascular dementia (VaD), and are limited by inclusion of a small number of studies and unexplained heterogeneity. Our review aimed to assess the risk of all-cause dementia, Alzheimer’s disease (AD) and VaD associated with smoking, and to identify potential effect modifiers.

**Methods and Findings:**

The PubMed, Embase, Cochrane Library and Psychinfo databases were searched to identify studies that provided risk estimates on smoking and incidence of dementia. A random-effects model was used to yield pooled results. Thirty-seven studies were included. Compared with never smokers, current smokers showed an increased risk of all-cause dementia (risk ratio (RR) 1.30, 95% confidence interval (CI) 1.18–1.45), AD (RR 1.40, 95% CI 1.13–1.73) and VaD (RR 1.38, 95% CI 1.15–1.66). For all-cause dementia, the risk increased by 34% for every 20 cigarettes per day (RR 1.34, 95% CI 1.25–1.43). Former smokers did not show an increased risk of all-cause dementia (RR 1.01, 95% CI 0.96–1.06), AD (RR 1.04, 95% CI 0.96–1.13) and VaD (RR 0.97, 95% CI 0.83–1.13). Subgroup analyses indicated that (1) the significantly increased risk of AD from current smoking was seen only in apolipoprotein E ε4 noncarriers; (2) current smokers aged 65 to 75 years at baseline showed increased risk of all-cause dementia and AD compared to those aged over 75 or under 65 years; and (3) sex, race, study location and diagnostic criteria difference in risk of dementia was not found.

**Conclusions:**

Smokers show an increased risk of dementia, and smoking cessation decreases the risk to that of never smokers. The increased risk of AD from smoking is more pronounced in apolipoprotein E ε4 noncarriers. Survival bias and competing risk reduce the risk of dementia from smoking at extreme age.

## Introduction

Dementia is a clinical state characterized by progressive deterioration in cognitive, functional and behavioral abilities [1]. It was estimated that the number of cases of dementia would reach around 81.1 million by 2040 [2]. Alzheimer’s disease (AD) and vascular dementia (VaD) could account for approximately 70% and 20% of dementia cases, respectively [1].

The global prevalence of smoking in the population aged more than 15 years is 31.1% and 6.2% for men and women in 2012, respectively [3]. The smoking-dementia relationship has been investigated by many studies [4–10]. A pooled analysis [11] found that mortality associated dementia was higher in smokers than that in never smokers. Nevertheless, researchers seem to be more interested in the association of smoking with risk of developing dementia. Up to date, two meta-analyses [12, 13] on smoking and risk of all-cause dementia, AD and VaD and two meta-analyses [14, 15] focusing on smoking and risk of AD have been published. These previous reviews reported that cigarette smoking might be a risk factor for developing dementia. However, several limitations as described below indicate that a more comprehensive meta-analysis is needed.

First, inclusion of a small number of studies was a limitation in previous reviews [12–15]. A 2014 meta-analysis [15] on smoking and risk of AD and a 2008 meta-analysis [12] on smoking and risk of all-cause dementia, AD and VaD involved 9 and 13 studies, respectively. Yet, more than 20 additional studies were published in the past several years. Second, previous meta-analyses [12–15] detected significant heterogeneity across studies, but they all did not identify the potential sources of heterogeneity. The unexplained heterogeneity raised questions regarding the reliability of pooled results. Third, a 2007 meta-analysis of 11 studies [13] and a 2008 meta-analysis of 13 studies [12] presented inconsistent results on the association of smoking with all-cause dementia and VaD. Thus, the association of smoking with all-cause dementia and VaD needs to be further investigated. Fourth, the potential dose–response pattern on smoking-dementia association and the modification effect of apolipoprotein E (APOE) ε4 allele on this association remain unclear. Finally, the results of a recent meta-analysis [14] by Cataldo et al on smoking and risk of AD may be biased by not classifying smokers to current and former smokers and the possibility of misclassification caused by inclusion of two large cohort studies [16, 17] using death certificates for AD diagnosis.

With those considerations above, our objectives for this study were: (1) to calculate the risk of all-cause dementia, AD and VaD for current versus never smokers, former versus never smokers and ever versus never smokers; (2) to identify potential effect modifiers of association between smoking and all-cause dementia, AD and VaD; (3) to explore the dose–response pattern of the association of smoking and dementia.

## Materials and Methods

### 1. Search strategy

This meta-analysis was conducted in accordance with PRISMA statement [18]. We conducted an electronic search of PubMed, Embase, Cochrane Library and Psychinfo from their inception to March 25, 2014, with limitation to human subjects imposed. We used the following search terms: “Alzheimer's disease”, “dementia”, “vascular dementia”, “cognitive impairment”, “cognitive decline”, “cognition”, “smoking”, “cigarette”, “cigarettes”, “nicotine” “tobacco” and “smoke”. We also reviewed the reference lists of identified studies and pertinent reviews for additional citations. We did not contact original authors through e-mails for extra data parameters.

### 2. Study selection

Studies were included if they (1) had a prospective cohort study design and were published in English or Chinese; (2) examined smoking status and identified that participants were free of dementia at baseline; and (3) reported minimum information necessary to obtain risk ratio (RR) on smoking and incident dementia (all-cause dementia, AD or VaD).

The process of study screening was independently conducted by two reviewers (GCZ and YW). An initial screening by scanning titles and abstracts was conducted to exclude irrelevant studies. We conducted a second screening by reading the full text to exclude unrelated articles. Any disagreement about eligibility of studies was resolved by consensus.

### 3. Data extraction

Two reviewers (GCZ and YW) independently extracted information. Discrepancies between two reviewers were settled by discussion. Collected information was presented as follows: last name of the first author, publication date, study location, mean age of participants at baseline, sex, race, number of cases and participants, number of current smokers, source of cohort, female proportion in study population, maximum length of follow-up, loss to follow-up rates, smoking category, outcome of interest, diagnostic criteria, the most fully adjusted risk estimates with corresponding 95% confidence interval (CI), and adjustment factors.

### 4. Statistical analysis and quality assessment

A random-effects model was used to yield summary effect size. We used I^2^ statistic to quantitatively describe heterogeneity across studies [19]. High heterogeneity existed when I^2^ is more than 75%, moderate heterogeneity when I^2^ ranged from 50% to 75% and low heterogeneity when I^2^ was less than 50%. To reflect the stability of our results and to identify potential sources of between-study variability, we conducted sensitivity analyses through three methods, namely ignoring a single study in turn, repeating our analyses through a fixed-effects model, and using various exclusion criteria. Where possible, subgroup analyses were also performed to explore underlying sources of heterogeneity. A *p*-value for heterogeneity between subgroups was calculated through meta-regression.

Given that all types of dementia are relatively uncommon events, the hazard ratio (HR) and odds ratio (OR) were roughly equal to RR [20, 21]. The HR and OR were therefore directly regarded as RR when RR was unavailable. We derived unadjusted RR from the corresponding exposure distribution when HR, OR and RR were unavailable. We used data from the report with the longest follow-up duration when multiple reports originated from the same population. If stratified results by smoking status (i.e., current and former) were separately reported, we summarized these stratum data through a random-effects model to produce an average estimate for ever smoking. Similarly, for two studies [4, 22] presenting measures by exposure level, we combined these risk estimates through a random-effects model to obtain the overall value for our meta-analysis. One study [5], whose authors only provided measures for men and women separately, was treated as two separate reports.

We conducted a dose-response analysis based on the method previously described by Orsini et al [23]. Given that the original researchers did not report person-year by exposure level, we approximately derived such data from mean duration of follow up and number of participants at each exposure level. We designated the midpoint of lower and upper boundaries as the assigned dose because all available data parameters of tobacco consumption were reported as range. Furthermore, if the highest range was open-ended, we considered that it shared the same width as the adjacent range. We fitted log-linear dose-response model to regress the log RR on the exposure level.

To reflect the impact of current smoking on the incidence of dementia at a population level, population attributable fraction (PAF) was calculated by the following formula: PAF = {P×(RR-1)/[P×(RR-1)+1]}. Here, P and RR denote the prevalence of current smoking and the summary RR, respectively. On the basis of included studies that reported prevalence of current smoking at baseline, we chose the median prevalence because the prevalence distribution was skewed.

We judged the methodological quality of included studies through the Newcastle–Ottawa quality assessment scale [24]. This tool could award a maximum of nine stars for each cohort study: four stars for the selection of study cohorts, two stars for the comparability of study groups and three stars for the ascertainment of outcome. If a study obtained six or more stars, it was considered to be of high quality.

Publication bias was tested with Begg’s test [25] and Egger’s test [26]. Data synthesis and analysis were performed via STATA software (version12.0, StataCorp, College Station, TX). Statistical significance level was set at *p*<0.05 under two-sided test unless otherwise specified.

## Results

### 1. Literature search

**Fig 1:**
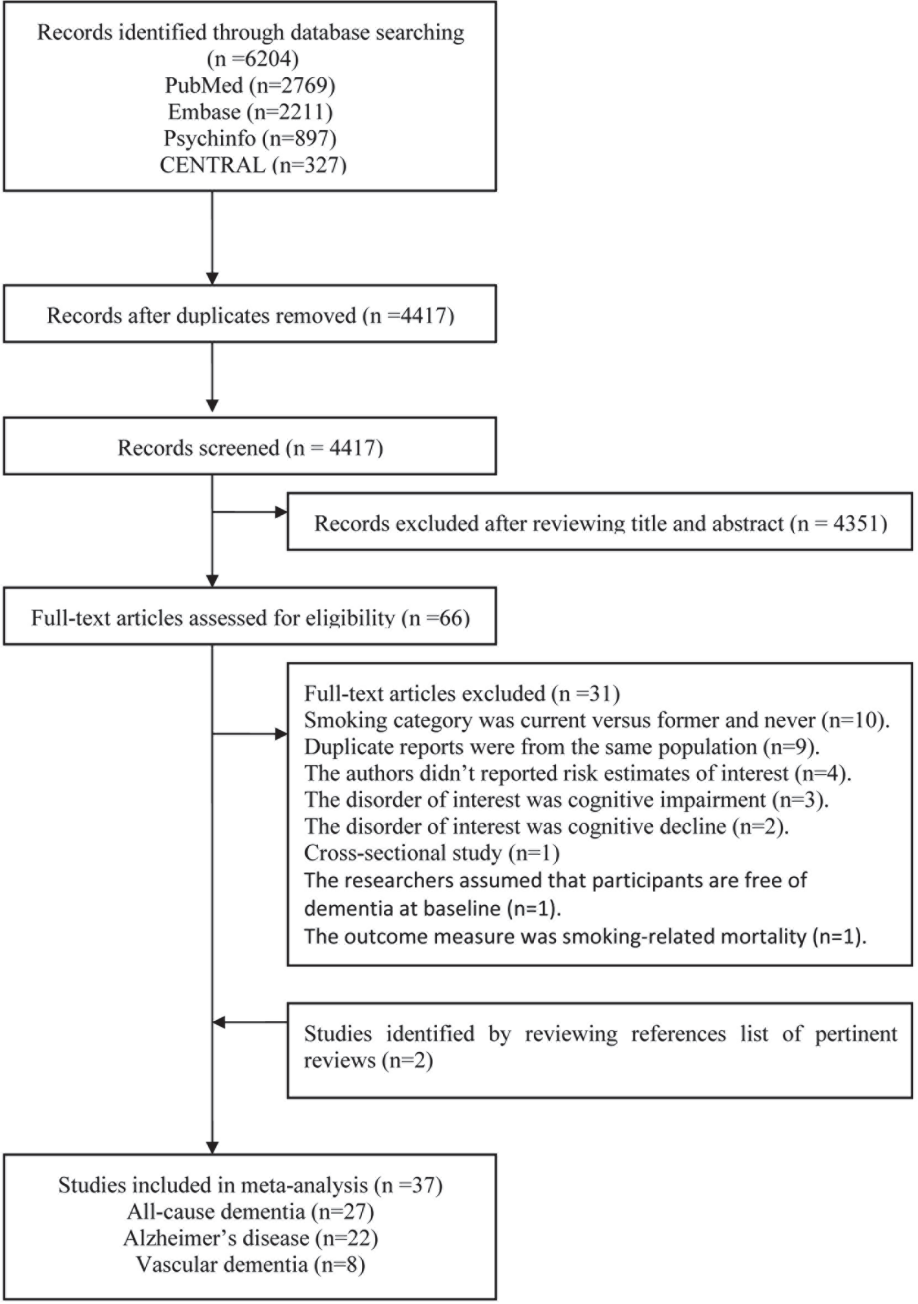
The flowchart of identifying relevant studies.

We identified 4,417 relevant citations after removing duplicates. A total of 4,351 citations were further excluded after reviewing their title and abstract. The remaining 66 citations were assessed in more detail for eligibility by reading the full text. Of these, 31 were excluded. We added 2 studies [27, 28] through the process of scanning the reference lists of pertinent reviews. Finally, 37 studies were used for the final data synthesis (Fig. 1).

### 2. Study characteristics

The main characteristics of included studies are shown in Table 1. Our meta-analysis involved 960,280 individuals, and documented 14,935 all-cause dementia cases, 5,816 AD cases, and 1,406 VaD cases during follow-up varying from 2 years [9] to 40 years [29]. The sample size of included studies ranged from 163 [30] to 848,505 [5]. The average score for methodological quality of included studies was 6.22, with 24 studies showing high quality (S1 Table). 29 studies reported information on loss to follow-up. Of these, 14 had low loss to follow-up rate (≦20%). All cohorts were derived from general populations except the two cohorts from hospitals [22, 31]. The mean age of participants at baseline ranged from 42.5 years [31] to 84.0 years [32]. Note that three studies [31, 33, 34] and one study [29] recruited volunteers and twins as their participants, respectively. 34 studies reported information about sex ratios. Of these, the proportion of women ranged from 0% [10, 22, 33–37] to 81.34% [32]. The overall proportion of women in these 34 studies was 42.12%.

**Table 1:** Characteristics of 37 included studies regarding smoking and risk of dementia.

Source	Female (%)	Sample size	Age^1^	Cases	Follow-up^2^ (y)	Smoking category	Outcome	Diagnosis criteria	Adjustment factors^4^
Bowen et al [42]	58.52	808	77.5	277	5	Current, former	Dementia	Unclear^3^	Age, sex, education, APOE ε4, BMI, alcohol, hypertension, diabetes, other
Zhou et al [67]	42.69	2019	72.2	132	5	Current, former	AD	NINCDS-ADRDA	Age, sex, education
Rusanen et al [4]	56.96	21123	58.0	Dementia:5376; AD:1136;VaD:416	30	Current, former	Dementia, AD, VaD	ICD-9-CM	Age, sex, education, BMI, alcohol, hypertension, diabetes, other
Ronnemaa et al [68]	0	2268^5^	50.0	Dementia:349; AD:127; VaD:81	40	Ever	Dementia, AD, VaD	Dementia: DSM-IV; AD:NINCDS-A RDA; VaD:ADDTC core criteria	Age, education
Ogunniyi et al [69]	69.02	1753	76.2	120	6	Ever	Dementia	ICD-10, DSM-III-R	None
Lin et al [33]	43.66	639	63.7	58	18	Current, former	Dementia	DSM-III-R	None
Kimm et al [5] (man)	0	490445	51.9	Dementia:3252; AD:1851; VaD:610	14	Current, former	Dementia, AD, VaD	Dementia: DSM-IV; AD:ICD-10; VaD: ICD-10	Age, alcohol
Kimm et al [5] (women)	100	358060	53.6	Dementia:3252; AD:1851;VaD:610	14	Current, former	Dementia, AD, VaD	Dementia: DSM-IV; AD:ICD-10; VaD: ICD-10	Age, alcohol
Gao et al [70]	71.45	1331	82.2	207	10	Current, former	Dementia	ICD-10, DSM-III-R	None
Chen et al [71]	NA	1238	>65.0	80	7.5	Current, former	Dementia	GMS-AGECAT, DSM-III	Age, sex
Brian et al [35]	0	12047	72.1	1271	13.4	Current, former	Dementia	ICD-9, ICD-10	None
Rusanen et al [6]	62.65	1449	50.6	Dementia:59; AD:46	26	Current, former	Dementia, AD	Dementia: DSM-IV; AD:NINCDS-ADRDA	APOE ε4, BMI, diabetes, other
Scarmeas et al [72]	68.78	1880	77.2	282	14	Ever	AD	NINCDS-ADRDA	None
Hassing et al [29]	69.00	1152	52.5	Dementia:312; AD:181; VaD:69	40	Ever	Dementia, AD, VaD	Dementia: DSM-III-R AD:NINCDS-ADRDA; VaD: NINDS-AIREN	None
Alonso et al [73]	57.47	1115	56.5	203	14	Current, former	Dementia	ICD-9	Age, sex, education, APOE ε4, BMI, hypertension, diabetes, other
Kivipelto et al [74]	61.25	1284	50.1	57	27	Ever	Dementia	DSM-IV	Age, sex, education, APOE ε4, BMI, diabetes, other
Dahl et al [75]	60.50	605	70.8	86	8	Ever	Dementia	DSM-IV	None
Beydoun et al [76]	36.53	2322	57.8	187	>20	Current, former	AD	NINCDS-ADRDA	None
Reitz et al [7]	61.46	6868	69.5	Dementia:706; AD:555; VaD:79	14	Current, former	Dementia, AD, VaD	Dementia: DSM-III-R; AD: NINCDS-ADRDA; VaD: NINDS-AIREN	Age, sex, education, alcohol
Laurin et al [36]	0	2588	76.9	240	7.8	Current, former	Dementia	DSM-III-R	None
Aggarwal et al [8]	61.90	1064	73.8	170	6.9	Current, former	AD	NINCDS-ADRDA	Age, sex, education, APOE ε4, other
Whitmer et al [31]	54.15	9217	42.5	713	9	Ever	Dementia	ICD-9	None
Rosengren et al [22]	0	7376	51.5	254	24	Current, former	Dementia	ICD-8, ICD-9, ICD-10	Age
Cherubini et al [77]	56.00	1033	75.5	58	NA	Current, former	Dementia	DSM-IV	None
Moffat et al [34]	0	574	66.3	Dementia:68; AD:43	37	Ever	Dementia, AD	Dementia: DSM-III-R; AD:NINCDS-ADRDA	Age, education, BMI, diabetes, other
Laurin et al [37]	0	2341	77.4	235	9	Current, former	Dementia	DSM-III-R	None
Juan et al [9]	NA	2820	66.9	Dementia:121; AD:84; VaD:16	2	Current, former	Dementia, AD, VaD	Dementia: DSM-III-R; AD:NINCDS-ADRDA; VaD: NINDS-AIREN	Age, sex, education, alcohol, other
Tyas et al [10]	0	3232	77.7	Dementia:297; AD:113; VaD:85	6	Current, former	Dementia, AD, VaD	Dementia: DSM-III-R; AD:NINCDS-ADRDA; VaD: ADDTC core criteria	Age, education, APOE ε4, alcohol, hypertension, other
Laurin et al [30]	65.80	163	78.8	52	5	Ever	Dementia	DSM-IV	None
Lindsay et al [78]	57.97	3973	73.3	194	5	Ever	AD	DSM-IV	Age, sex, education
Tyas et al [79]	62.40	644	74.0	36	5	Ever	AD	NINCDS-ADRDA	None
Wang et al [32]	81.34	343	84.0	Dementia:46; AD:34	3	Ever	Dementia, AD	DSM-III-R with minor modification	Age, sex, education
Merchant et al [54]	68.74	1062	75.4	142	>2	Current, former	AD	NINCDS-ADRDA	None
Launer et al [28]	NA	12843	>65.0	Dementia:400; AD:277	≧5	Current, former	Dementia, AD	Dementia: DSM-III-R; AD:NINCDS-ADRDA;	Age, sex, education, other
Broe et al [27]	49.50	299	83.4	Dementia:47; AD:29	3	Current, former^6^	Dementia, AD	Dementia: DSM-III-R; AD:NINCDS-ADRDA;	Age, sex, education
Yoshitake et al [80]	59.69	826	73.6	AD:42; VaD:50	7	Ever	AD, VaD	AD:NINCDS-ADRDA; VaD: NINDS-AIREN	Age
Letenneur et al [81]	58.25	3770	>65.0	79	NA	Ever, current, former	AD	NINCDS-ADRDA	Age, sex, education, other
Hebert et al [82]	55.75	513	>65.0	76	3	Ever	AD	NINCDS-ADRDA	Age, sex, education

APOE ε4, apolipoprotein E ε4; NA, not available; BMI, body mass index; IGT, impaired glucose tolerance; NINCDS-ADRDA, DSM-III-R, Diagnostic and Statistical Manual of Mental Disorders, third edition Revised; DSM-IV, Diagnostic and Statistical Manual of Mental Disorders, fourth edition; NINDS-AIREN, National Institute of Neurological Disorders and Stroke-Association Internationale pour la Recherche et l'Enseignement en Neurosciences; GMS-AGECAT, Geriatric Mental State-the Automated Geriatric Examination for Computer Assisted Taxonomy; ICD-8, International Classification of Diseases, Eighth Revision; ADDTC core criteria, Alzheimer’s Disease Diagnostic and Treatment Centers core criteria.

^1^ Value refers to mean age of participants at baseline.

^2^ Value is expressed as maximum.

^3^ Dementia was determined by a battery of neuropsychological measures and a standardized neurological examination.

^4^ The term “other” in the “Adjustment factors” column refers to all the confounders except age, sex, education, APOE ε4, BMI, diabetes, alcohol and hypertension.

^5^ Value refers to sample size at baseline.

^6^ The risk estimates were available just for former smoking and the risk of all-cause dementia and AD.

Adjusted RRs were available for 22 studies and most adjusted for age (21 studies) and sex (15 studies). The diagnostic criteria for dementia varied across studies, but Diagnostic and Statistical Manual of Mental Disorders, Fourth Edition (DSM-IV) [38] and Diagnostic and Statistical Manual of Mental Disorders, Third Edition Revised (DSM-III-R) [39] were the most commonly used for dementia diagnosis; National Institute of Neurological and Communicative Diseases and Stroke-Alzheimer’s Disease and Related Disorders Association (NINCDS-ADRDA) [40] was the most used for AD diagnosis, and National Institute of Neurological Disorders and Stroke-Association Internationale pour la Recherche et l'Enseignement en Neurosciences (NINDS-AIREN) [41] was the most used for VaD diagnosis.

### 3. Current smoking and risk of all-cause dementia, AD and VaD

**Fig 2:**
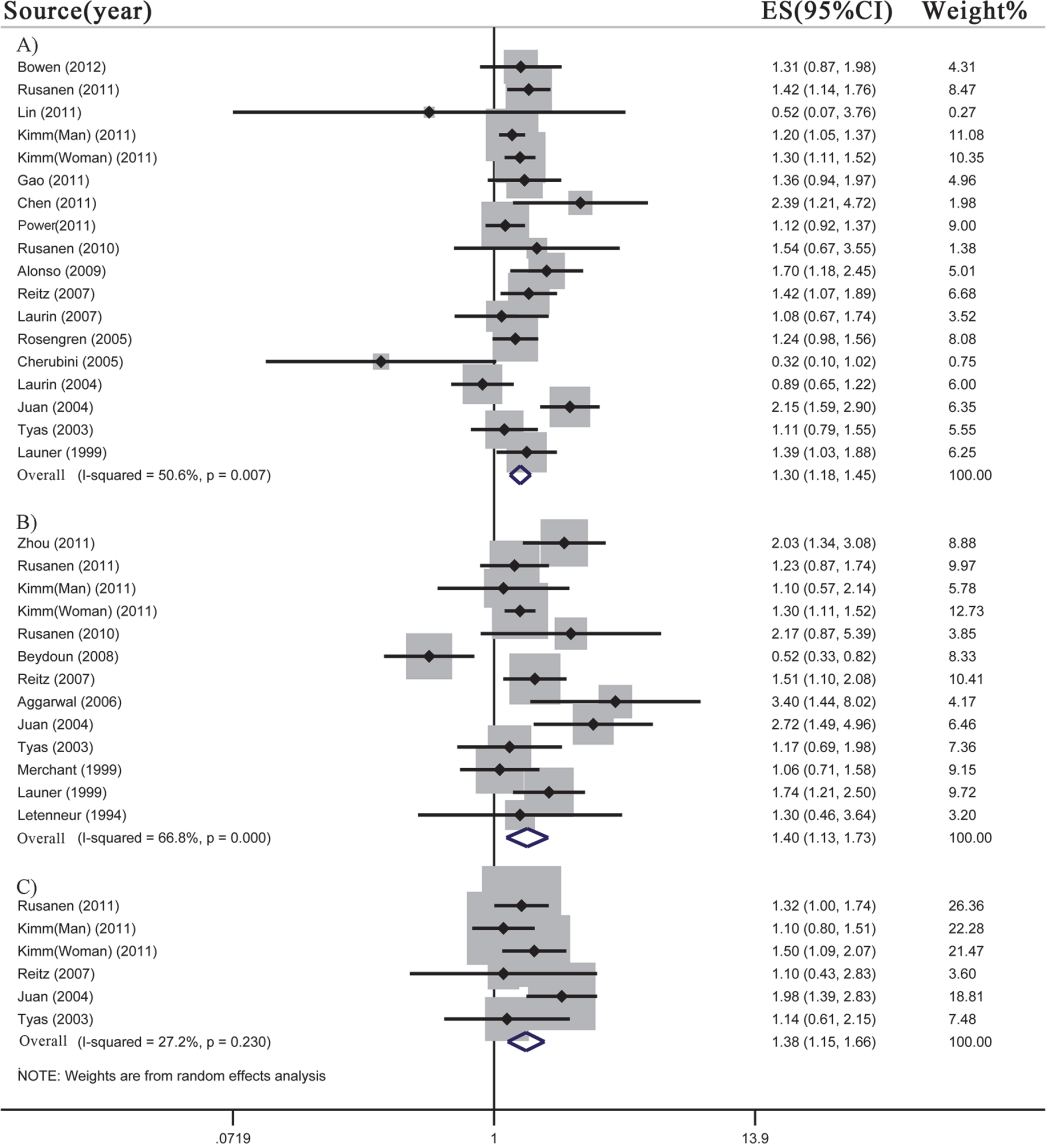
Meta-analysis for current smoking and risk of A) all-cause dementia, B) Alzheimer’s disease and C) vascular dementia.

Our meta-analyses involved 937,392 subjects for all-cause dementia, 907,077 subjects for AD, and 882,548 subjects for VaD. Current smokers had significantly increased risk of all-cause dementia (n = 17 studies; RR 1.30, 95% CI 1.18–1.45), AD (n = 12 studies; RR 1.40, 95% CI 1.13–1.73) and VaD (n = 5 studies; RR 1.38, 95% CI 1.15–1.66) compared to never smokers (Fig. 2). There was evidence of low heterogeneity of pooled RR of VaD (I^2^ = 27.2%; *p* = 0.23), and moderate heterogeneity of pooled RR of all-cause dementia (I^2^ = 50.6%; *p*<0.01) and AD (I^2^ = 66.8%; *p*<0.01).

### 4. Former smoking and risk of all-cause dementia, AD and VaD

**Fig 3:**
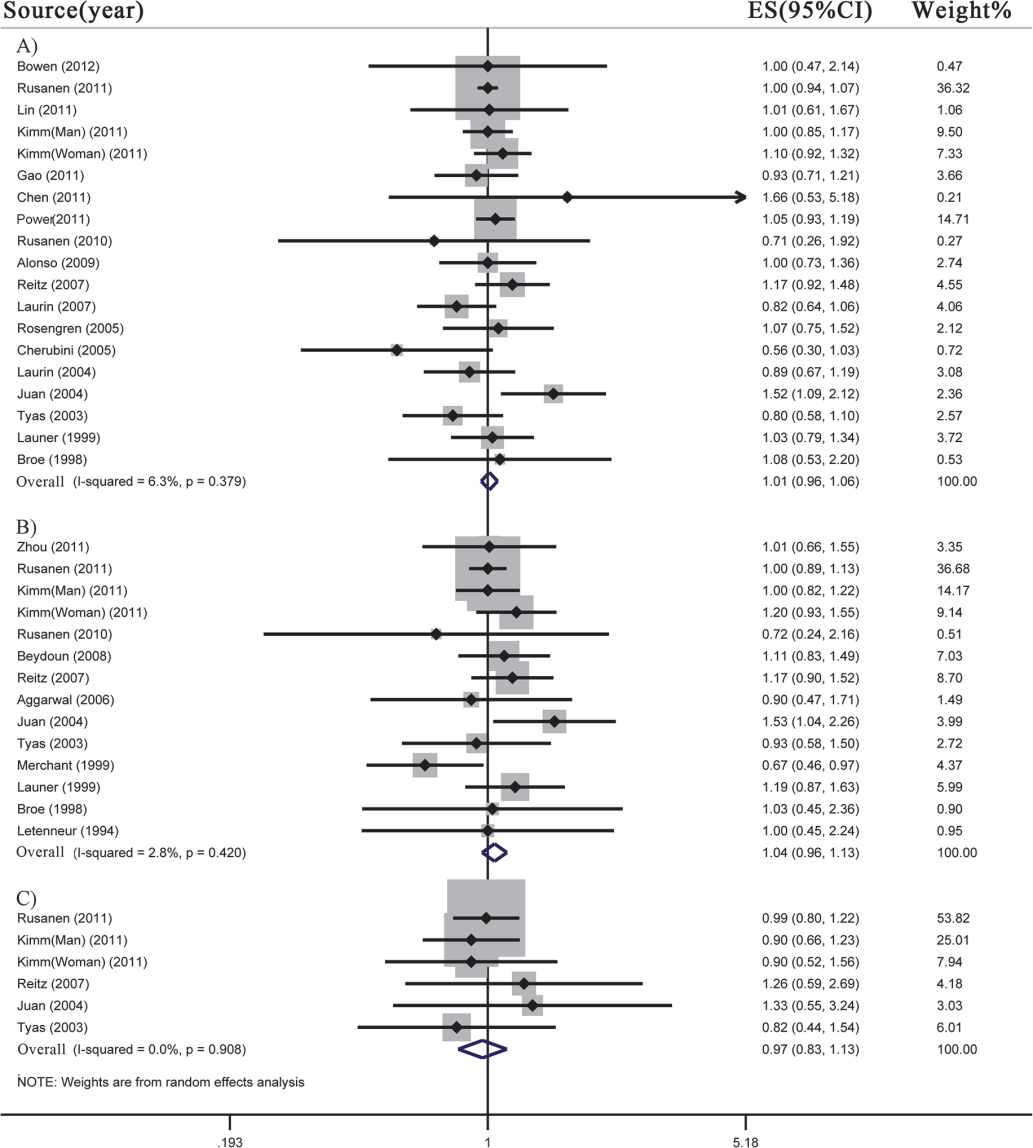
Meta-analysis for former smoking and risk of A) all-cause dementia, B) Alzheimer’s disease and C) vascular dementia.

Our meta-analyses included 937,691 subjects for all-cause dementia, 907,376 subjects for AD, and 882,548 subjects for VaD. Former smokers did not show increased risk of all-cause dementia (n = 18 studies; RR 1.01, 95% CI 0.96–1.06), AD (n = 13 studies; RR 1.04, 95% CI 0.96–1.13) and VaD (n = 5 studies; RR 0.97, 95% CI 0.83–1.13) compared to never smokers (Fig. 3). Low heterogeneity was observed for all-cause dementia (I^2^ = 6.3%; *p* = 0.38), AD (I^2^ = 2.8%; *p* = 0.42), and VaD (I^2^ = 0.0%; *p* = 0.91).

### 5. Ever smoking and risk of all-cause dementia, AD and VaD

**Fig 4:**
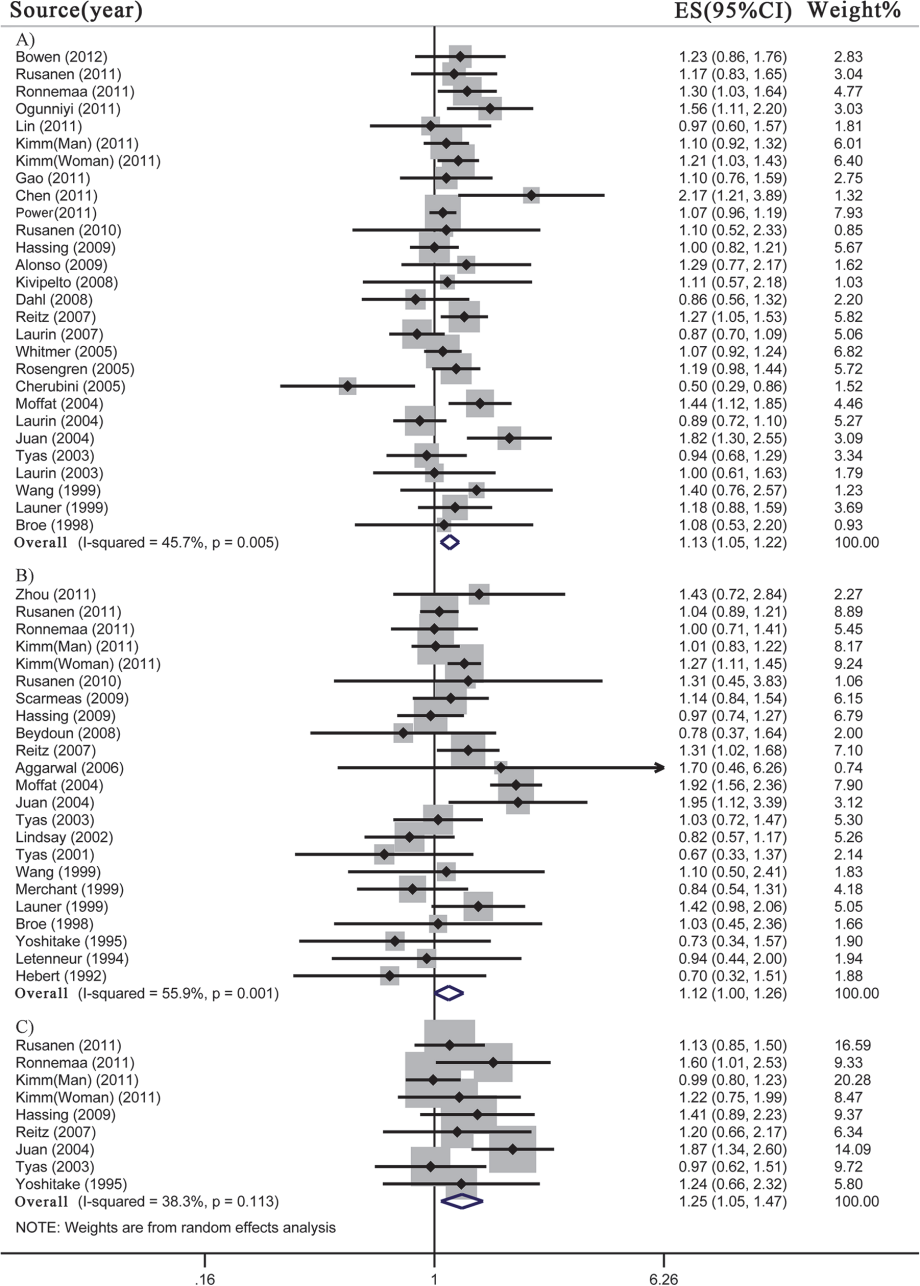
Meta-analysis for ever smoking and risk of A) all-cause dementia, B) Alzheimer’s disease and C) vascular dementia.

Our meta-analyses included 955,050 subjects for all-cause dementia, 919,549 subjects for AD, and 886,794 subjects for VaD. Ever smoking showed significantly increased risk of all-cause dementia (n = 27 studies; RR 1.13, 95% CI 1.05–1.22) and VaD (n = 8 studies; RR 1.25, 95% CI 1.05–1.47) compared to never smokers, with low heterogeneity (all-cause dementia, I^2^ = 45.7%, *p*<0.01; VaD, I^2^ = 38.3%, *p* = 0.11) (Fig. 4). However, the increased risk of AD was of marginal significance (n = 22 studies; RR 1.12, 95% CI 1.00–1.26), with moderate heterogeneity (I^2^ = 55.9%; *p*<0.01).

### 6. Sensitivity analyses

Sensitivity analyses by exclusion of any single study in turn did not materially change the pooled risk estimates of all-cause dementia, AD and VaD for current and former smokers (Table 2). Furthermore, when we examined the effect of various exclusion criteria (see Table 2 for details of various exclusion criteria) on the summary results and repeated our analysis through the fixed-effects model, the aforementioned initial associations still remained. Similar results were observed for the association between ever smoking and all-cause dementia. However, the associations of ever smoking with AD and VaD were not stable. Specifically, the pooled RR of AD for ever smoking ranged from 1.10 (95% CI 0.97–1.26) to 1.14 (95% CI 1.02–1.29) in the analysis of omitting a single study in turn. Moreover, the analysis through the fixed-effects model produced a statistically significant RR of 1.17 (95% CI 1.09–1.24). For ever smoking and VaD, the result by exclusion of the study by Juan et al [9] was marginal (RR 1.13, 95% CI 0.99–1.29).

**Table 2:** Sensitivity analyses of smoking and dementia

Categories	Ever versus never smoking			Current versus never smoking			Former versus never smoking		
n	RR (95% CI)	I^2^ (%)	n	RR (95% CI)	I^2^ (%)	n	RR (95% CI)	I^2^ (%)	
All-cause dementia									
*Pooling model*									
Random effects	27	1.13 (1.05–1.22)	45.7	17	1.30 (1.18–1.45)	50.6	18	1.01 (0.96–1.06)	6.3
Fixed effects	27	1.12 (1.07–1.17)	45.7	17	1.28 (1.20–1.37)	50.6	18	1.01 (0.96–1.06)	6.3
*Analysis of all studies except*									
Studies basing on hospital	25	1.13 (1.04–1.23)	48.9	16	1.31 (1.17–1.47)	53.4	17	1.01 (0.95–1.07)	11.0
Studies recruiting volunteers as participants	24	1.13 (1.04–1.22)	46.8	16	1.31 (1.18–1.45)	52.4	17	1.01 (0.95–1.07)	11.5
Alzheimer’s disease									
*Pooling model*									
Random effects	22	1.12 (1.00–1.26)	55.9	12	1.40 (1.13–1.73)	66.8	13	1.04 (0.96–1.13)	2.8
Fixed effects	22	1.17 (1.09–1.24)	55.9	12	1.35 (1.21–1.49)	66.8	13	1.04 (0.97–1.12)	2.8
*Analysis of all studies except*									
Studies recruiting volunteers as participants	21	1.09 (1.00–1.18)	16.6	12	1.40 (1.13–1.73)	66.8	13	1.04 (0.96–1.13)	2.8
Studies using other criteria than NINCDS-ADRDA	18	1.14 (0.97–1.35)	56.3	10	1.50 (1.09–2.06)	74.1	11	1.06 (0.93–1.22)	12.4
Vascular dementia									
*Pooling model*									
Random effects	8	1.25 (1.05–1.47)	38.3	5	1.38 (1.15–1.66)	27.2	5	0.97 (0.83–1.13)	0.0
Fixed effects	8	1.21 (1.07–1.37)	38.3	5	1.38 (1.19–1.60)	27.2	5	0.97 (0.83–1.13)	0.0

CI, confidence interval; NINCDS-ADRDA, National Institute of Neurological and Communicative Diseases and Stroke-Alzheimer’s Disease and Related Disorders Association; RR, risk ratio.

Excluding studies relying on other diagnostic criteria than NINCDS-ADRDA did not change the association of smoking and AD. The moderate heterogeneity of the pooled RR of AD decreased to 16.6% (I^2^ value) when we excluded studies that recruited volunteers. We did not identify any source of heterogeneity for the pooled RR of all-cause dementia and AD through sensitivity analyses.

### 7. Subgroup analyses

**Fig 5:**
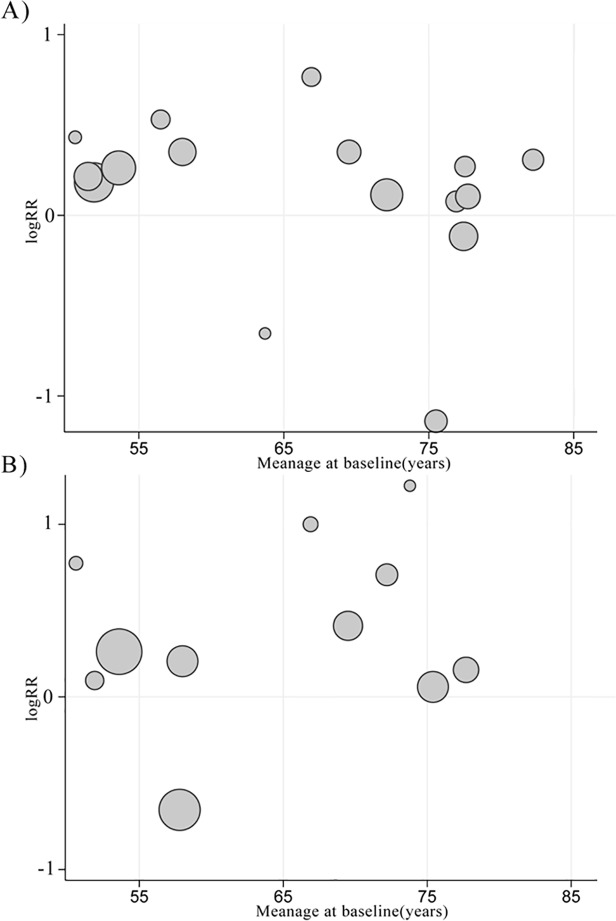
LogRR of A) all-cause dementia and B) Alzheimer’s disease by the mean age at baseline. Each circle represents an individual study. The area of circle is proportional to the inverse variance of logrr. RR, risk ratio.

The results of subgroup analyses on association of smoking with all-cause dementia and AD are summarized in Tables 3 and 4. Current smokers aged 65 to 75 years at baseline showed increased risk of all-cause dementia and AD compared to those aged over 75 or under 65 years (Fig. 5), despite the fact that the difference between these subgroups was not significant (all *p* for heterogeneity>0.05). Current smokers without APOE ε4 allele showed a significantly increased risk of AD (RR 2.01, 95% CI 1.34–3.03), whereas the association between current smoking and AD in APOE ε4 carriers was not significant (RR 1.51; 95% CI 0.69–3.28).

**Table 3:** Subgroup analyses of smoking and all-cause dementia.

Subgroup	Ever versus never smoking				Current versus never smoking				Former versus never smoking			
n	RR (95% CI)	I^2^ (%)	*p* ^1^	n	RR (95% CI)	I^2^ (%)	*p* ^1^	n	RR (95% CI)	I^2^ (%)	*p* ^1^	
*All studies*	27	1.13 (1.05–1.22)	45.7	-	17	1.30 (1.18–1.45)	50.6	-	18	1.01 (0.96–1.06)	6.3	-
*Sex*												
Man	8	1.08 (0.97–1.21)	56.4	0.54	6	1.15 (1.05–1.26)	0.0	0.24	6	0.98 (0.89–1.06)	7.2	0.32
Woman	1	1.21 (1.03–1.43)	-		1	1.30 (1.11–1.52)	-		1	1.10 (0.92–1.32)	-	
*Study location*												
North American	11	1.06 (0.95–1.17)	26.7	0.17	8	1.24 (1.06–1.46)	32.2	0.59	8	0.98 (0.92–1.03)	0.0	0.18
Europe	10	1.11 (0.97–1.27)	42.2		5	1.29 (1.04–1.60)	38.2		5	1.02 (0.84–1.23)	26.9	
Asia	3	1.38 (1.08–1.75)	70.7		3	1.52 (1.18–1.97)	80.3		3	1.14 (0.95–1.36)	44.9	
*Race*												
White	13	1.11 (1.01–1.21)	27.1	0.64	6	1.24 (1.05–1.47)	36.7	0.93	7	1.05 (0.95–1.15)	0.0	0.81
Yellow	6	1.14 (0.94–1.37)	75.0		6	1.30 (1.07–1.58)	72.7		6	1.00 (0.86–1.16)	52.6	
Black	2	1.32 (0.94–1.86)	45.7		1	1.36 (0.94–1.97)	-		1	0.93 (0.71–1.21)	-	
*Sample size*												
≧1000	20	1.13 (1.04–1.22)	53.4	0.78	15	1.31 (1.17–1.46)	55.3	0.86	15	1.01 (0.95–1.08)	21.7	0.94
<1000	7	1.19 (1.02–1.39)	0.0		2	1.26 (0.84–1.89)	0.0		3	1.03 (0.71–1.47)	0.0	
*Maximum duration of follow-up*												
≧10y	13	1.15 (1.08–1.22)	0.0	0.53	9	1.27 (1.19–1.37)	0.0	0.82	9	1.02 (0.97–1.07)	0.0	0.32
<10y	13	1.14 (0.99–1.31)	59.1		7	1.35 (1.04–1.76)	71.3		8	0.99 (0.83–1.17)	39.1	
*Loss to follow-up rate*												
>20%	9	1.04 (0.91–1.19)	37.0	0.49	5	1.22 (0.99–1.51)	41.8	0.97	5	0.98 (0.93–1.04)	0.0	0.44
≦20%	11	1.10 (0.99–1.24)	45.3		8	1.23 (1.03–1.46)	40.7		9	1.03 (0.93–1.14)	1.9	
*Mean age at baseline*												
<65y	10	1.13 (1.05–1.21)	0.0	0.13	6	1.29 (1.18–1.40)	0.0	0.21	6	1.01 (0.96–1.07)	0.0	0.03
65∼75y	5	1.25 (1.03–1.52)	72.5		3	1.49 (1.02–2.17)	84.3		3	1.17 (0.97–1.41)	0.0	
≧75y	10	1.01 (0.88–1.19)	50.8		6	1.08 (0.87–1.34)	37.7		7	0.85 (0.75–0.97)	0.0	
*Diagnosis criteria of dementia*												
DSM-Ⅳ	8	1.08 (0.93–1.24)	37.2	0.59	3	1.22 (1.00–1.47)	50.6	0.64	4	1.00 (0.86–1.16)	18.7	0.93
DSM-Ⅲ-R	11	1.12 (0.98–1.29)	61.9		8	1.30 (1.04–1.61)	63.5		8	0.99 (0.86–1.15)	45.5	
*Adjusted risk estimates*												
Yes	16	1.24 (1.15–1.32)	0.0	<0.01	11	1.39 (1.25–1.54)	42.4	0.03	12	1.02 (0.97–1.08)	0.0	0.22
No	11	1.00 (0.90–1.11)	45.0		6	1.04 (0.84–1.29)	35.9		6	0.93 (0.82–1.06)	26.9	
*Adjustment for confounders*												
*Body mass index (or Diabetes mellitus)* ^*2*^												
Yes	6	1.29 (1.10–1.51)	0.0	0.25	4	1.46 (1.23–1.72)	0.0	0.34	4	1.00 (0.94–1.06)	0.0	0.67
No	21	1.11 (1.02–1.21)	53.2		13	1.27(1.12–1.43)	57.7		14	1.01 (0.94–1.10)	24.5	
*Hypertension*												
Yes	4	1.12 (0.93–1.34)	0.0	0.96	4	1.38(1.18–1.60)	0.0	0.65	4	0.99 (0.93–1.06)	0.0	0.45
No	23	1.13 (1.05–1.23)	52.0		13	1.28(1.13–1.46)	57.2		14	1.03 (0.95–1.11)	16.5	
*Alcohol*												
Yes	6	1.21 (1.08–1.36)	35.6	0.25	6	1.37(1.20–1.57)	57.1	0.39	6	1.05(0.95–1.16)	40.1	0.36
No	21	1.10 (1.00–1.20)	45.2		11	1.23(1.04–1.46)	47.0		12	0.98(0.91–1.07)	0.0	
*Education*												
Yes	12	1.28 (1.16–1.40)	0.0	0.02	7	1.48(1.27–1.72)	40.4	0.04	8	1.04 (0.94–1.16)	25.0	0.61
No	15	1.06 (0.97–1.16)	48.8		10	1.19(1.06–1.34)	35.9		10	1.00 (0.93–1.07)	0.0	

CI, confidence interval; RR, risk ratio; DSM-IV, Diagnostic and Statistical Manual of Mental Disorders, Fourth Edition; DSM-III-R, Diagnostic and Statistical Manual of Mental Disorders, Third Edition Revised.

^1^ *P* for heterogeneity between subgroups with meta-regression.

^2^ Note that among selected studies for body mass index and diabetes mellitus, researchers adjusted these two confounders in tandem. Thus, the results of subgroup analyses regarding body mass index and diabetes mellitus are identified.

**Table 4:** Subgroup analyses of smoking and Alzheimer’s disease.

Subgroup	Ever versus never smoking				Current versus never smoking				Former versus never smoking			
n	RR (95% CI)	I^2^ (%)	*p* ^1^	n	RR (95% CI)	I^2^ (%)	*p* ^1^	n	RR (95% CI)	I^2^ (%)	*p* ^1^	
*All studies*	22	1.12 (1.00–1.26)	55.9	-	12	1.40 (1.13–1.73)	66.8	-	13	1.04 (0.96–1.13)	2.8	-
*Sex*												
Man	5	1.34 (0.94–1.91)	86.3	0.85	3	1.51 (0.83–2.74)	59.4	0.81	3	1.07 (0.80–143)	34.3	0.47
Woman	2	1.27 (1.12–1.43)	0.0		2	1.32 (1.14–1.53)	0.0		2	1.16 (0.94–1.44)	0.0	
*Study location*												
North American	10	1.03 (0.81–1.31)	75.1	0.71	5	1.13 (0.72–1.76)	76.9	0.32	5	0.96 (0.83–1.11)	18.5	0.25
Europe	7	1.14 (0.99–1.32)	0.0		4	1.62 (1.29–2.03)	0.0		4	1.15 (0.95–1.39)	0.0	
Asia	4	1.20 (0.97–1.49)	53.4		3	1.64 (1.14–2.36)	67.1		3	1.13 (0.95–1.35)	27.7	
*Race*												
White	10	1.07 (0.94–1.23)	4.5	0.44	4	1.62 (1.29–2.03)	0.0	0.68	5	1.14 (0.95–1.38)	0.0	0.75
Yellow	5	1.17 (0.98–1.40)	45.7		4	1.53 (1.14–2.06)	58.1		4	1.11 (0.95–1.28)	14.3	
*Sample size*												
≧1000	16	1.11 (1.01–1.21)	22.6	0.60	12	1.40 (1.13–1.73)	66.8	-	12	1.05 (0.96–1.14)	10.3	0.98
<1000	6	1.00 (0.61–1.64)	73.5		-	-	-		1	1.03 (0.45–2.36)	-	
*Maximum duration of follow-up*												
≧10y	9	1.17 (1.01–1.37)	72.2	0.53	5	1.16 (0.87–1.56)	71.3	0.07	5	1.05 (0.96–1.14)	0.0	0.81
<10y	11	1.08 (0.88–1.32)	27.7		5	1.92 (1.43–2.59)	40.3		6	1.14 (0.95–1.37)	0.0	
*Loss to follow-up rate*												
>20%	11	1.00 (0.90–1.12)	0.0	0.14	6	1.32 (0.85–2.05)	79.7	0.67	6	0.99 (0.85–1.14)	23.1	0.48
≦20%	6	1.17 (1.00–1.37)	0.0		3	1.57 (1.19–2.06)	26.1		4	1.08 (0.89–1.32)	0.0	
*Apolipoprotein E ε4 carrier*												
Yes	4	0.94 (0.58–1.52)	51.7	0.46	4	1.51 (0.69–3.28)	60.2	0.29	4	0.83 (0.51–1.36)	37.4	0.81
No	4	1.25 (0.74–2.12)	21.2		4	2.01 (1.34–3.03)	23.3		4	0.93 (0.62–1.40)	29.1	
*Mean age at baseline*												
<65y	6	1.08 (0.98–1.20)	22.9	0.28	4	1.09 (0.76–1.57)	74.5	0.08	4	1.03 (0.94–1.13)	0.0	0.11
65∼75y	8	1.24 (0.91–1.69)	73.1		4	2.04 (1.47–2.83)	42.6		4	1.18 (0.98–1.42)	0.0	
≧75y	5	1.04 (0.85–1.26)	0.0		2	1.10 (0.80–1.51)	0.0		3	0.79 (0.60–1.04)	0.0	
*Adjusted risk estimates*												
Yes	17	1.18 (1.03–1.35)	59.4	0.12	10	1.55 (1.30–1.83)	39.0	0.01	11	1.06 (0.98–1.15)	0.0	0.25
No	5	0.97 (0.81–1.15)	0.0		2	0.75 (0.37–1.50)	81.2		2	0.87 (0.53–1.43)	77.0	
*Adjustment for confounders*												
*Body mass index (or Diabetes mellitus)* ^*2*^												
Yes	3	1.39 (0.82–2.36)	90.8	0.12	2	1.39 (0.88–2.19)	23.5	0.89	2	1.00 (0.88–1.12)	0.0	0.44
No	19	1.09 (0.99–1.20)	22.0		10	1.40 (1.10–1.78)	71.3		11	1.07 (0.97–1.19)	9.3	
*Hypertension*												
Yes	2	0.96 (0.69–1.34)	28.6	0.37	2	1.21 (0.91–1.62)	0.0	0.58	2	1.00 (0.89–1.12)	0.0	0.38
No	20	1.14 (1.01–1.30)	55.4		10	1.45 (1.13–1.87)	71.9		11	1.08 (0.97–1.20)	10.4	
*Alcohol*												
Yes	5	1.16 (1.00–1.35)	59.2	0.64	5	1.37 (1.16–1.62)	27.0	0.98	5	1.08 (0.97–1.20)	21.4	0.39
No	17	1.08 (0.91–1.29)	57.4		7	1.45 (0.92–2.28)	79.5		8	0.99 (0.85–1.16)	0.0	
*Education*												
Yes	14	1.20 (1.00–1.44)	64.1	0.16	8	1.66 (1.34–2.05)	37.8	0.05	9	1.06 (0.96–1.16)	0.0	0.49
No	8	1.05 (0.92–1.20)	29.9		4	1.05 (0.72–1.55)	75.0		4	0.99 (0.82–1.20)	44.2	

CI, confidence interval; RR, risk ratio.

^1^ P for heterogeneity between subgroups with meta-regression.

^2^ Note that among selected studies for body mass index and diabetes mellitus, researchers adjusted these two confounders in tandem. Thus, the results of subgroup analyses regarding body mass index and diabetes mellitus are identified.

The association between former smoking and all-cause dementia and AD persisted in all subgroups, with no evidence of heterogeneity between subgroups from meta-regression, apart from the subgroup for all-cause dementia stratified by the mean age of participants (*p* = 0.03). In the analysis stratified by adjusted risk estimates, we found evidence of significant heterogeneity between subgroups for the association between current smoking and all-cause dementia (*p* = 0.03) and AD (*p* = 0.01). Sex, study location, race and several key study characteristics, including sample size, mean duration of follow-up and loss to follow-up rate, were not the source of heterogeneity for pooled RR of all-cause dementia and AD for current, former and ever smoking.

Additionally, we found no significant difference between the subgroup using DSM-IV criteria and the subgroup using DSM-III-R criteria. The small number of studies of ever smoking and VaD precluded our interpretation to the results of subgroup analyses (S2 Table). We did not perform subgroup analysis with respect to current smoking, former smoking and VaD since limited studies [4, 5, 7, 9, 10] were available.

### 8. Dose–response analysis

**Fig 6:**
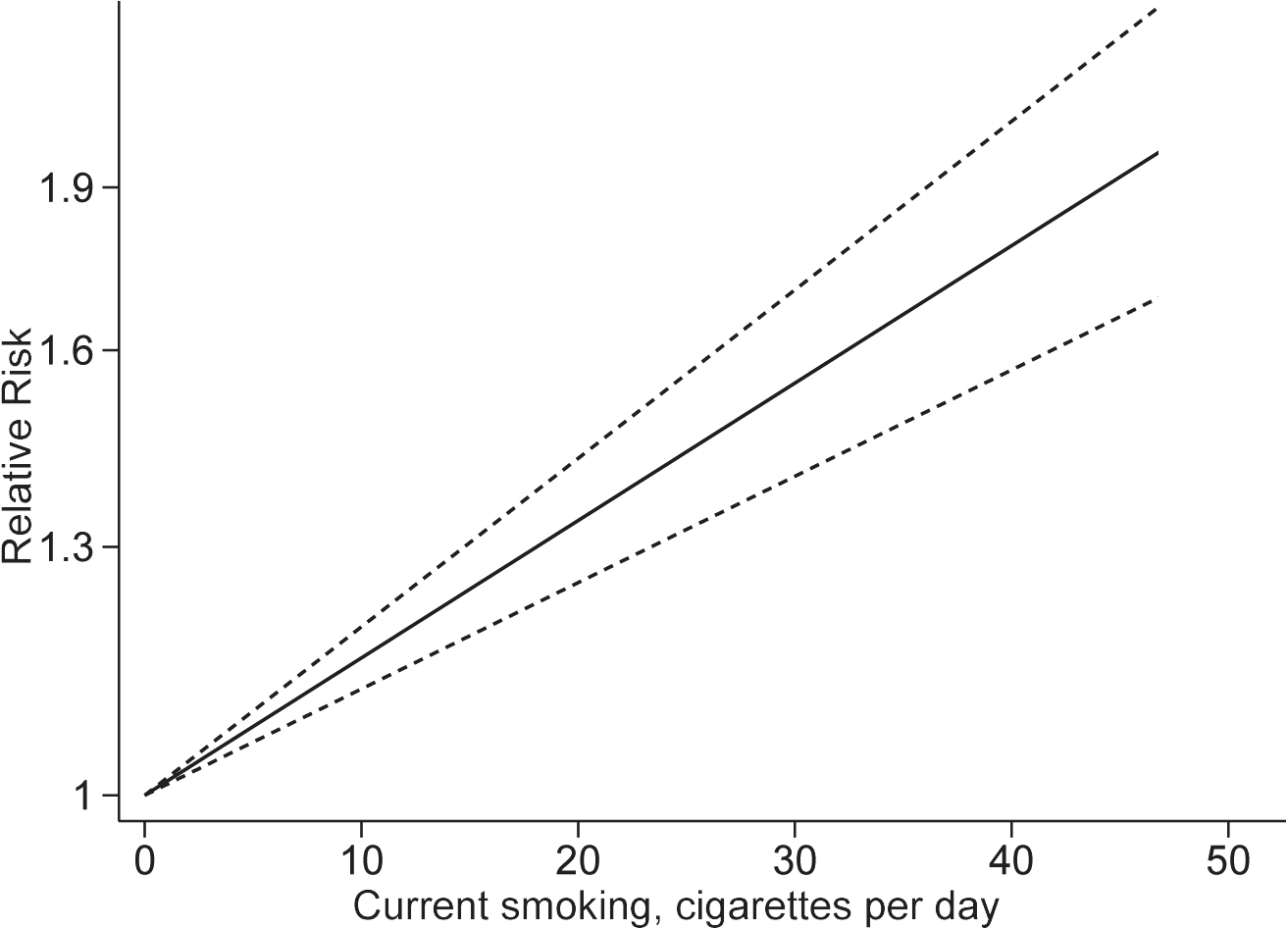
The linear dose–response relationship plot between current smoking and all-cause dementia. The solid line represents the linear trend and lines with short dashes represent its’ 95% confidence interval.

Only two studies [4, 22] were included in the dose–response analysis of current smoking and all-cause dementia. Under the fixed-effect dose–response meta-regression model, the risk of dementia significantly increased by 34% for every 20 cigarettes per day (RR 1.34, 95% CI 1.25–1.43; *p* for nonlinearity = 0.36, goodness-of-fit chi^2^ = 5.91, p = 0.43; Fig. 6). For current smoking and incident AD and VaD, only one study was available, which showed a dose–response relationship [4].

### 9. PAF calculation

Fifteen studies reported the prevalence of current smoking in their study population. The prevalence of current smoking ranged from 8.8% (95% CI 6.8%–10.7%) [42] to 34.5% (95% CI 34.4%–34.6%) [5], with the median value of 20.7% (95% CI 20.2%–21.3%) [4]. The PAF of all-cause dementia, AD, and VaD for current smoking was 5.8% (95% CI 3.5%–8.7%), 7.6% (95% CI 2.6%–13.5%) and 7.3% (95% CI 2.9%–12.3%), respectively.

### 10. Publication bias

No evidence of publication bias was found for any association by Begg’s test and Egger’s test (all *p*>0.05).

## Discussion

The present meta-analysis identified that current smokers consistently showed increased risks of all-cause dementia, AD, and VaD compared with never smokers, while former smokers did not show increased risks. We also found that ever smokers showed increased risks of all-cause dementia and VaD, but the associations were weaker than that for current smokers. The increased risk of AD in ever smokers was marginal.

A diagnosis of dementia can never be valid and definitive without subsequent histopathological confirmation and this is challenging for researchers who rely on clinical diagnoses [43]. Accordingly, participants generally received a clinical diagnosis of dementia on the basis of various diagnostic classification systems. Several diagnostic criteria of dementia including DSM-IV criteria, DSM-III-R criteria, and NINCDS-ADRDA criteria are widely used. However, some studies observed that the prevalence of dementia varied remarkably when researchers relied on different diagnostic criteria [44, 45]. A large number of studies included in the present study allowed us to examine the effects of different diagnostic classification systems on the risk of dementia through subgroup and sensitivity analysis. We found that the diagnostic criteria of dementia did not modify the smoking-dementia association.

Previous studies indicated that the incidence of dementia rises with increasing age [46–49]. However, in subgroup analyses stratified by age at baseline, we observed an unanticipated decline in the risk of all-cause dementia and AD among current and ever smokers aged from 65–75 to over 75 years, most notably for current smokers. A possible interpretation for above phenomenon is that the risk of all-cause dementia and AD should rise with increasing age, but survival bias [50] and competing risk [51] apparently reduce the risk of dementia from smoking at extreme age. In fact, several previous studies [52, 53] have observed similar phenomena. Hernan and colleagues [53] observed that the weighted average RR of incident AD gradually decreased with increasing age. Similarly, Taylor and colleagues [52] found a positive association among individuals aged less than 50 years but an inverse association among those aged more than 50 years when investigating the relationship between smoking increasing allele and smoking initiation. Generally, survival bias will be introduced when researchers recruit older smokers into their study cohorts at baseline, and competing risk will occur if a smoker dies of smoking-associated events (e.g., lung cancer) before receiving a diagnosis of dementia during follow-up [51]. Given these facts, future epidemiologic studies on “bad” exposures and age-related outcomes should take into account the influence of survival bias and competing risk when making interpretations to their results.

The modification effect of APOE ε4 allele on the association between smoking and dementia remains controversial. As suggested by our subgroup analyses, the association of current smoking and increased risk of AD remained significant only in APOE ε4 noncarriers. Our findings are consistent with results of previous prospective studies [7, 8, 54]. The underlying mechanisms for the aforementioned results are unclear. APOE ε4 carriers are at an elevated risk of AD [55–57]. A potential explanation for our results is that the increased risk of AD due to APOE ε4 allele makes the increased risk due to other risk factors insubstantial. We could not assess the combined effects of smoking and APOE ε4 allele on all-cause dementia and VaD because of few individual studies exploring it. Further research is warranted to clarify the modification effect of APOE ε4 allele on association of smoking with dementia.

In the present study, smokers were grouped into current, former and ever smokers. Our meta-analyses observed increased risk of dementia in ever but not former smokers. Considering ever smokers consist of current and former smokers, this phenomenon may be attributable to the influence of current smokers. Indeed, as shown by our forest plots, risks of dementia in ever smokers were lower than those in current smokers but higher than those in former smokers.

The potential biological mechanisms regarding smoking and increased risk of dementia have been proposed [58, 59]. In brief, smoking could disturb the balance between generation and reduction of oxidants and free radical species. The consequent overload of oxidants and free radical species triggers oxidative stress (OS). Increased OS contributes to formation of senile plaque and neurofibrillary tangles, signifying the occurrence of dementia. In addition, increased risk of dementia resulting from smoking may be realized through cardiovascular disease (CVD) because smoking-related OS has been hypothesized as an underlying mechanism for CVD [60], and CVD contribute to an increased risk of dementia [61, 62].

The heterogeneity of the studies regarding adjustment of the risk estimates for underlying confounders is a possible limitation of our review. Smoking is associated with other dietary, lifestyle and social factors, such as higher intake of alcohol, lower body weight, higher blood pressure and lower education level. The subgroup analyses stratified by adjustment for body mass index, hypertension, alcohol and education showed consistent results on current smoking and all-cause dementia, as well as former smoking and all-cause dementia and AD, but the results for current smoking and AD only remained significant in subgroups with adjustment for alcohol and education. In addition, the limited number of studies adjusting for body mass index and hypertension could be subject to over-adjustment because both could be mediators in the causal pathway between smoking and dementia. Considering these facts, we could not completely rule out the possibility that the inadequate control for various underlying confounders may bias our findings. A promising and useful approach to reduce confounding bias is Mendelian randomization [63, 64], and it can assess the potential causal associations between exposures and end points. Fortunately, genome-wide association studies have identified that the CHRNA3 rs1051730 genotype is a common genetic marker strongly associated with smoking behavior [65, 66]. Thus, it raises a possibility of using Mendelian randomization to explore the nature of smoking-dementia association.

Our study also has several strengths. Inclusion of only prospective cohort studies can avoid recall bias and chicken–egg question occurring in case-control studies. We included a large number of studies, so we had enough statistical power to identify smoking-dementia association. Subgroup analyses promote a better understanding of smoking-dementia association. The stability of pooled results as evidenced by sensitivity analyses indicates the robustness of our findings. No statistical evidence of publication bias and inclusion of studies that treated smoking as a covariate further support the robustness of our findings. In addition, we explored the dose–response pattern between smoking and risk of all-cause dementia for the first time, despite the fact that only two studies were available.

## Conclusions

Our meta-analysis indicates that smokers show an increased risk of dementia, and quitting smoking decreases the risk to that of never smokers. The increased risk of AD from smoking is more pronounced in APOE ε4 noncarriers. Both survival bias and competing risk reduce the risk of dementia from smoking at extreme age. The smoking-dementia relationship could not be modified by sex, race, study location and the diagnostic criteria of dementia. Future study is warranted to clarify the modification effect of APOE ε4 allele on association of smoking with dementia.

## Supporting Information

S1 PRISMA Checklist PRISMA checklist. (DOC)

S1 Dataset Original data associated with the present study. (XLS)

S1 Table The results of quality assessment. (DOC)

S2 Table Subgroup analyses of smoking and vascular dementia. (DOC)

## References

[1] Reitz C, Brayne C, Mayeux R. Epidemiology of Alzheimer disease. Nature reviews Neurology. 2011;7: 137–152. 10.1038/nrneurol.2011.2 21304480 PMC3339565

[2] Organization World Health. Dementia: a public health priority: World Health Organization;2012.

[3] Ng M, Freeman MK, Fleming TD, Robinson M, Dwyer-Lindgren L, Thomson B, et al Smoking prevalence and cigarette consumption in 187 countries, 1980–2012. JAMA: the journal of the American Medical Association. 2014;311: 183–192. 10.1001/jama.2013.284692 24399557

[4] Rusanen M, Kivipelto M, Quesenberry CP Jr, Zhou J, Whitmer RA. Heavy smoking in midlife and long-term risk of Alzheimer disease and vascular dementia. Archives of internal medicine. 2011;171: 333–339. 10.1001/archinternmed.2010.393 20975015

[5] Kimm H, Lee PH, Shin YJ, Park KS, Jo J, Lee Y, et al Mid-life and late-life vascular risk factors and dementia in Korean men and women. Archives of gerontology and geriatrics. 2011;52: e117–122. 10.1016/j.archger.2010.09.004 20932588

[6] Rusanen M, Rovio S, Ngandu T, Nissinen A, Tuomilehto J, Soininen H, et al Midlife smoking, apolipoprotein E and risk of dementia and Alzheimer's disease: a population-based cardiovascular risk factors, aging and dementia study. Dementia and geriatric cognitive disorders. 2010;30: 277–284. 10.1159/000320484 20847559

[7] Reitz C, den Heijer T, van Duijn C, Hofman A, Breteler MM. Relation between smoking and risk of dementia and Alzheimer disease: the Rotterdam Study. Neurology. 2007;69: 998–1005. 17785668 10.1212/01.wnl.0000271395.29695.9a

[8] Aggarwal NT, Bienias JL, Bennett DA, Wilson RS, Morris MC, Schneider JA, et al The relation of cigarette smoking to incident Alzheimer's disease in a biracial urban community population. Neuroepidemiology. 2006;26: 140–146. 16493200 10.1159/000091654

[9] Juan D, Zhou DH, Li J, Wang JY, Gao C, Chen M. A 2-year follow-up study of cigarette smoking and risk of dementia. European journal of neurology: the official journal of the European Federation of Neurological Societies. 2004;11: 277–282. 15061830 10.1046/j.1468-1331.2003.00779.x

[10] Tyas SL, White LR, Petrovitch H, Webster Ross G, Foley DJ, Heimovitz HK, et al Mid-life smoking and late-life dementia: the Honolulu-Asia Aging Study. Neurobiology of aging. 2003;24: 589–596. 12714116 10.1016/s0197-4580(02)00156-2

[11] Batty GD, Russ TC, Starr JM, Stamatakis E, Kivimaki M. Modifiable cardiovascular disease risk factors as predictors of dementia death: pooling of ten general population-based cohort studies. Journal of negative results in biomedicine. 2014;13: 8 10.1186/1477-5751-13-8 24886432 PMC4036694

[12] Peters R, Poulter R, Warner J, Beckett N, Burch L, Bulpitt C. Smoking, dementia and cognitive decline in the elderly, a systematic review. BMC geriatrics. 2008;8: 36 10.1186/1471-2318-8-36 19105840 PMC2642819

[13] Anstey KJ, Von Sanden C, Salim A, O'Kearney R. Smoking as a risk factor for dementia and cognitive decline: A meta-analysis of prospective studies. American journal of epidemiology. 2007;166: 367–378. 17573335 10.1093/aje/kwm116

[14] Cataldo JK, Prochaska JJ, Glantz SA. Cigarette smoking is a risk factor for Alzheimer's disease: An analysis controlling for tobacco industry affiliation. Journal of Alzheimer's Disease. 2010;19: 465–480. 10.3233/JAD-2010-1240 20110594 PMC2906761

[15] Beydoun MA, Beydoun HA, Gamaldo AA, Teel A, Zonderman AB, Wang Y. Epidemiologic studies of modifiable factors associated with cognition and dementia: systematic review and meta-analysis. BMC public health. 2014;14: 643 10.1186/1471-2458-14-643 24962204 PMC4099157

[16] Doll R, Peto R, Boreham J, Sutherland I. Smoking and dementia in male British doctors: prospective study. BMJ (Clinical research ed). 2000;320: 1097–1102. 10775216 10.1136/bmj.320.7242.1097 PMC27350

[17] Hirayama T. Large cohort study on the relation between cigarette smoking and senile dementia without cerebrovascular lesions. Tobacco Control. 1992;1: 176.

[18] Moher D, Liberati A, Tetzlaff J, Altman DG. Preferred reporting items for systematic reviews and meta-analyses: the PRISMA statement. BMJ (Clinical research ed). 2009;339: b2535 10.1136/bmj.b2535 19622551 PMC2714657

[19] Higgins JP, Thompson SG, Deeks JJ, Altman DG. Measuring inconsistency in meta-analyses. BMJ (Clinical research ed). 2003;327: 557–560. 12958120 10.1136/bmj.327.7414.557 PMC192859

[20] Greenland S. Quantitative methods in the review of epidemiologic literature. Epidemiologic reviews. 1987; 9: 1–30. 3678409 10.1093/oxfordjournals.epirev.a036298

[21] Siristatidis C, Sergentanis TN, Kanavidis P, Trivella M, Sotiraki M, Mavromatis I, et al Controlled ovarian hyperstimulation for IVF: impact on ovarian, endometrial and cervical cancer—a systematic review and meta-analysis. Human reproduction update. 2013; 19: 105–123. 10.1093/humupd/dms051 23255514

[22] Rosengren A, Skoog I, Gustafson D, Wilhelmsen L. Body mass index, other cardiovascular risk factors, and hospitalization for dementia. Archives of internal medicine. 2005;165: 321–326. 15710796 10.1001/archinte.165.3.321

[23] Orsini N, Bellocco R, Greenland S. Generalized least squares for trend estimation of summarized dose–response data. The Stata Journal. 2006; 6(1):40–57.

[24] Wells G, Shea B, O’connell D, Peterson J, Welch V, Losos M, et al. The Newcastle-Ottawa Scale (NOS) for assessing the quality of nonrandomised studies in meta-analyses. 2000.

[25] Begg CB and Mazumdar M. Operating characteristics of a rank correlation test for publication bias. Biometrics. 1994;50: 1088–1101. 7786990

[26] Egger M, Davey Smith G, Schneider M, Minder C. Bias in meta-analysis detected by a simple, graphical test. BMJ (Clinical research ed). 1997;315: 629–634. 9310563 10.1136/bmj.315.7109.629 PMC2127453

[27] Broe GA, Creasey H, Jorm AF, Bennett HP, Casey B, Waite LM, et al Health habits and risk of cognitive impairment and dementia in old age: a prospective study on the effects of exercise, smoking and alcohol consumption. Australian and New Zealand journal of public health. 1998; 22: 621–623. 9744220 10.1111/j.1467-842x.1998.tb01449.x

[28] Launer LJ, Andersen K, Dewey ME, Letenneur L, Ott A, Amaducci LA, et al Rates and risk factors for dementia and Alzheimer's disease: results from EURODEM pooled analyses. EURODEM Incidence Research Group and Work Groups. European Studies of Dementia. Neurology. 1999;52: 78–84. 9921852 10.1212/wnl.52.1.78

[29] Hassing LB, Dahl AK, Thorvaldsson V, Berg S, Gatz M, Pedersen NL, et al Overweight in midlife and risk of dementia: a 40-year follow-up study. International journal of obesity (2005). 2009;33: 893–898. 10.1038/ijo.2009.104 19506566 PMC3025291

[30] Laurin D, Verreault R, Lindsay J, Dewailly E, Holub BJ. Omega-3 fatty acids and risk of cognitive impairment and dementia. Journal of Alzheimer's disease: JAD. 2003;5: 315–322. 14624027 10.3233/jad-2003-5407

[31] Whitmer RA, Gunderson EP, Barrett-Connor E, Quesenberry CP Jr, Yaffe K. Obesity in middle age and future risk of dementia: a 27 year longitudinal population based study. BMJ (Clinical research ed). 2005;330: 1360 15863436 10.1136/bmj.38446.466238.E0 PMC558283

[32] Wang HX, Fratiglioni L, Frisoni GB, Viitanen M, Winblad B. Smoking and the occurrence of Alzheimer's disease: cross-sectional and longitudinal data in a population-based study. American journal of epidemiology. 1999;149: 640–644. 10192311 10.1093/oxfordjournals.aje.a009864

[33] Lin FR, Metter EJ, O'Brien RJ, Resnick SM, Zonderman AB, Ferrucci L. Hearing loss and incident dementia. Archives of neurology. 2011;68: 214–220. 10.1001/archneurol.2010.362 21320988 PMC3277836

[34] Moffat SD, Zonderman AB, Metter EJ, Kawas C, Blackman MR, Harman SM, et al Free testosterone and risk for Alzheimer disease in older men. Neurology. 2004;62: 188–193. 14745052 10.1212/wnl.62.2.188

[35] Power BD, Alfonso H, Flicker L, Hankey GJ, Yeap BB, Almeida O. Body adiposity in later life and the incidence of dementia: the health in men study. PloS one. 2011;6: e17902 10.1371/journal.pone.0017902 21464984 PMC3064574

[36] Laurin D, Masaki KH, White LR, Launer LJ. Ankle-to-brachial index and dementia: the Honolulu-Asia Aging Study. Circulation. 2007;116: 2269–2274. 17967779 10.1161/CIRCULATIONAHA.106.686477

[37] Laurin D, Masaki KH, Foley DJ, White LR, Launer LJ. Midlife dietary intake of antioxidants and risk of late-life incident dementia: the Honolulu-Asia Aging Study. American journal of epidemiology. 2004;159: 959–967. 15128608 10.1093/aje/kwh124

[38] Association AP. Diagnostic and Statistical Manual of Mental Disorders. 4th edn Washington, DC: American Psychiatric Association 1994.

[39] Association AP. Diagnostic and Statistical Manual of Mental Disorders, Third Edition, Revised. Washington, DC: American Psychiatric Association 1987.

[40] McKhann G, Drachman D, Folstein M, Katzman R, Price D, Almeida O. Clinical diagnosis of Alzheimer's disease: report of the NINCDS-ADRDA Work Group under the auspices of Department of Health and Human Services Task Force on Alzheimer's Disease. Neurology. 1984;34: 939–944. 6610841 10.1212/wnl.34.7.939

[41] Roman GC, Tatemichi TK, Erkinjuntti T, Cummings JL, Masdeu JC, Garcia JH, et al Vascular dementia: diagnostic criteria for research studies. Report of the NINDS-AIREN International Workshop. Neurology. 1993;43: 250–260. 8094895 10.1212/wnl.43.2.250

[42] Bowen ME. A prospective examination of the relationship between physical activity and dementia risk in later life. American journal of health promotion: AJHP. 2012;26: 333–340. 10.4278/ajhp.110311-QUAN-115 22747314

[43] Karantzoulis S, Galvin JE. Distinguishing Alzheimer's disease from other major forms of dementia. Expert Rev Neurother. 2011;11: 1579–1591. 10.1586/ern.11.155 22014137 PMC3225285

[44] Erkinjuntti T, Ostbye T, Steenhuis R, Hachinski V. The effect of different diagnostic criteria on the prevalence of dementia. The New England journal of medicine. 1997;337: 1667–1674. 9385127 10.1056/NEJM199712043372306

[45] Pohjasvaara T, Erkinjuntti T, Vataja R, Kaste M. Dementia three months after stroke. Baseline frequency and effect of different definitions of dementia in the Helsinki Stroke Aging Memory Study (SAM) cohort. Stroke; a journal of cerebral circulation. 1997;28: 785–792. 9099197 10.1161/01.str.28.4.785

[46] Vardarajan BN, Faber KM, Bird TD, Bennett DA, Rosenberg R, Boeve BF, et al Age-specific incidence rates for dementia and Alzheimer disease in NIA-LOAD/NCRAD and EFIGA families: National Institute on Aging Genetics Initiative for Late-Onset Alzheimer Disease/National Cell Repository for Alzheimer Disease (NIA-LOAD/NCRAD) and Estudio Familiar de Influencia Genetica en Alzheimer (EFIGA). JAMA neurology. 2014;71: 315–323. 10.1001/jamaneurol.2013.5570 24425039 PMC4000602

[47] Jorm AF, Jolley D. The incidence of dementia: a meta-analysis. Neurology. 1998;51: 728–733. 9748017 10.1212/wnl.51.3.728

[48] Gao S, Hendrie HC, Hall KS, Hui S. The relationships between age, sex, and the incidence of dementia and Alzheimer disease: a meta-analysis. Archives of general psychiatry. 1998;55: 809–815. 9736007 10.1001/archpsyc.55.9.809

[49] Katz MJ, Lipton RB, Hall CB, Zimmerman ME, Sanders AE, Verghese J, et al Age-specific and sex-specific prevalence and incidence of mild cognitive impairment, dementia, and Alzheimer dementia in blacks and whites: a report from the Einstein Aging Study. Alzheimer disease and associated disorders. 2012; 26: 335–343. 10.1097/WAD.0b013e31823dbcfc 22156756 PMC3334445

[50] Hernan MA, Alonso A, Logroscino G. Cigarette smoking and dementia: potential selection bias in the elderly. Epidemiology (Cambridge, Mass). 2008;19: 448–450. 10.1097/EDE.0b013e31816bbe14 18414087

[51] Chang CC, Zhao Y, Lee CW, Ganguli M. Smoking, death, and Alzheimer disease: a case of competing risks. Alzheimer disease and associated disorders. 2012; 26: 300–306. 10.1097/WAD.0b013e3182420b6e 22185783 PMC3321062

[52] Taylor AE, Munafo MR. Commentary: Does mortality from smoking have implications for future Mendelian randomization studies? International journal of epidemiology. 2014;43: 1483–1486. 10.1093/ije/dyu151 25125581 PMC4190520

[53] Hernan MA, Alonso A, Logroscino G. Cigarette smoking and dementia: Potential selection bias in the elderly. Epidemiology (Cambridge, Mass). 2008;19: 448–450. 10.1097/EDE.0b013e31816bbe14 18414087

[54] Merchant C, Tang MX, Albert S, Manly J, Stern Y, Mayeux R. The influence of smoking on the risk of Alzheimer's disease. Neurology. 1999;52: 1408–1412. 10227626 10.1212/wnl.52.7.1408

[55] Chuang YF, Hayden KM, Norton MC, Tschanz J, Breitner JC, Welsh-Bohmer KA, et al Association between APOE epsilon4 allele and vascular dementia: The Cache County study. Dementia and geriatric cognitive disorders. 2010;29: 248–253. 10.1159/000285166 20375505 PMC2865397

[56] Koponen S, Taiminen T, Kairisto V, Portin R, Isoniemi H, Hinkka S, et al APOE-epsilon4 predicts dementia but not other psychiatric disorders after traumatic brain injury. Neurology. 2004;63: 749–750. 15326261 10.1212/01.wnl.0000134603.57107.2f

[57] Huriletemuer, Wang B, Wang J, Wang G, Zhang C, Zhao S, et al APOE epsilon4 is a high-risk factor for Alzheimer's disease in the Mongolian population. Journal of the neurological sciences. 2010;288: 167–169. 10.1016/j.jns.2009.08.058 19819468

[58] Durazzo TC, Mattsson N, Weiner MW. Smoking and increased Alzheimer's disease risk: A review of potential mechanisms. Alzheimer's & dementia: the journal of the Alzheimer's Association. 2014;10: S122–145. 10.1016/j.jalz.2014.04.009 PMC4098701 24924665

[59] Burke A, Fitzgerald GA. Oxidative stress and smoking-induced vascular injury. Progress in cardiovascular diseases. 2003;46: 79–90. 12920701 10.1016/s0033-0620(03)00076-8

[60] Ambrose JA, Barua RS. The pathophysiology of cigarette smoking and cardiovascular disease: an update. Journal of the American College of Cardiology. 2004;43: 1731–1737. 15145091 10.1016/j.jacc.2003.12.047

[61] Paciaroni M, Bogousslavsky J. Connecting cardiovascular disease and dementia: further evidence. Journal of the American Heart Association. 2013; 2: e000656 10.1161/JAHA.113.000656 24351703 PMC3886737

[62] Newman AB, Fitzpatrick AL, Lopez O, Jackson S, Lyketsos C, Jagust W, et al Dementia and Alzheimer's disease incidence in relationship to cardiovascular disease in the Cardiovascular Health Study cohort. Journal of the American Geriatrics Society. 2005; 53: 1101–1107. 16108925 10.1111/j.1532-5415.2005.53360.x

[63] Zoccali C, Testa A, Spoto B, Tripepi G, Mallamaci F. Mendelian randomization: a new approach to studying epidemiology in ESRD. American journal of kidney diseases: the official journal of the National Kidney Foundation. 2006; 47: 332–341. 16431263 10.1053/j.ajkd.2005.10.027

[64] Didelez V, Sheehan N. Mendelian randomization as an instrumental variable approach to causal inference. Statistical methods in medical research. 2007;16: 309–330. 17715159 10.1177/0962280206077743

[65] Kaur-Knudsen D, Nordestgaard BG, Bojesen SE. CHRNA3 genotype, nicotine dependence, lung function and disease in the general population. The European respiratory journal. 2012;40: 1538–1544. 10.1183/09031936.00176811 22441734

[66] Tobacco and Genetics Consortium. Genome-wide meta-analyses identify multiple loci associated with smoking behavior. Nature genetics. 2010;42: 441–447. 10.1038/ng.571 20418890 PMC2914600

[67] Zhou R, Deng J, Zhang M, Zhou HD, Wang YJ. Association between bone mineral density and the risk of Alzheimer's disease. Journal of Alzheimer's disease: JAD. 2011;24: 101–108. 10.3233/JAD-2010-101467 21187587

[68] Ronnemaa E, Zethelius B, Lannfelt L, Kilander L. Vascular risk factors and dementia: 40-year follow-up of a population-based cohort. Dementia and geriatric cognitive disorders. 2011;31: 460–466. 10.1159/000330020 21791923

[69] Ogunniyi A, Lane KA, Baiyewu O, Gao S, Gureje O, Unverzagt FW, et al Hypertension and incident dementia in community-dwelling elderly Yoruba Nigerians. Acta neurologica Scandinavica. 2011;124: 396–402. 10.1111/j.1600-0404.2011.01491.x 21303353 PMC3099146

[70] Gao S, Nguyen JT, Hendrie HC, Unverzagt FW, Hake A, Smith-Gamble V, et al Accelerated weight loss and incident dementia in an elderly African-American cohort. Journal of the American Geriatrics Society. 2011; 59: 18–25. 10.1111/j.1532-5415.2010.03169.x 21054328 PMC3020982

[71] Chen R, Hu Z, Wei L, Ma Y, Liu Z, Copeland JR. Incident dementia in a defined older Chinese population. PloS one. 2011; 6: e24817US. 10.1371/journal.pone.0024817 21966372 PMC3179466

[72] Scarmeas N, Luchsinger JA, Schupf N, Brickman AM, Cosentino S, Tang MX, et al Physical activity, diet, and risk of Alzheimer disease. JAMA: the journal of the American Medical Association. 2009;302: 627–637. 10.1001/jama.2009.1144 19671904 PMC2765045

[73] Alonso A, Mosley TH Jr, Gottesman RF, Catellier D, Sharrett AR, Coresh J. Risk of dementia hospitalisation associated with cardiovascular risk factors in midlife and older age: the Atherosclerosis Risk in Communities (ARIC) study. Journal of neurology, neurosurgery, and psychiatry. 2009; 80: 1194–1201. 10.1136/jnnp.2009.176818 19692426 PMC2783764

[74] Kivipelto M, Rovio S, Ngandu T, Kareholt I, Eskelinen M, Winblad B, et al Apolipoprotein E epsilon4 magnifies lifestyle risks for dementia: a population-based study. Journal of cellular and molecular medicine. 2008; 12: 2762–2771. 10.1111/j.1582-4934.2008.00296.x 18318693 PMC3828889

[75] Dahl AK, Lopponen M, Isoaho R, Berg S, Kivela SL. Overweight and obesity in old age are not associated with greater dementia risk. Journal of the American Geriatrics Society. 2008; 56: 2261–2266. 10.1111/j.1532-5415.2008.01958.x 19093925

[76] Beydoun MA, Lhotsky A, Wang Y, Dal Forno G, An Y, Metter EJ, et al Association of adiposity status and changes in early to mid-adulthood with incidence of Alzheimer's disease. American journal of epidemiology. 2008;168: 1179–1189. 10.1093/aje/kwn229 18835864 PMC2582058

[77] Cherubini A, Martin A, Andres-Lacueva C, Di Iorio A, Lamponi M, Mecocci P, et al Vitamin E levels, cognitive impairment and dementia in older persons: the InCHIANTI study. Neurobiology of aging. 2005;26: 987–994. 15748776 10.1016/j.neurobiolaging.2004.09.002

[78] Lindsay J, Laurin D, Verreault R, Hebert R, Helliwell B, Hill GB, et al Risk factors for Alzheimer's disease: a prospective analysis from the Canadian Study of Health and Aging. American journal of epidemiology. 2002;156: 445–453. 12196314 10.1093/aje/kwf074

[79] Tyas SL, Manfreda J, Strain LA, Montgomery PR. Risk factors for Alzheimer's disease: a population-based, longitudinal study in Manitoba, Canada. International journal of epidemiology. 2001;30: 590–597. 11416089 10.1093/ije/30.3.590

[80] Yoshitake T, Kiyohara Y, Kato I, Ohmura T, Iwamoto H, Nakayama K, et al Incidence and risk factors of vascular dementia and Alzheimer's disease in a defined elderly Japanese population: the Hisayama Study. Neurology. 1995;45: 1161–1168. 7783883 10.1212/wnl.45.6.1161

[81] Letenneur L, Dartigues JF, Commenges D, Barberger-Gateau P, Tessier JF, Orgogozo JM. Tobacco consumption and cognitive impairment in elderly people. A population-based study. Annals of epidemiology. 1994; 4: 449–454. 7804499 10.1016/1047-2797(94)90004-3

[82] Hebert LE, Scherr PA, Beckett LA, Funkenstein HH, Albert MS, Chown MJ, et al Relation of smoking and alcohol consumption to incident Alzheimer's disease. American journal of epidemiology. 1992;135: 347–355. 1550089 10.1093/oxfordjournals.aje.a116296

