# Correction: Smoking Is Associated with an Increased Risk of Dementia: A Meta-Analysis of Prospective Cohort Studies with Investigation of Potential Effect Modifiers

**DOI:** 10.1371/journal.pone.0126169

**Published:** 2015-04-13

**Authors:** The PLOS ONE Staff

There is an error in affiliation 1 for authors Guochao Zhong and Yi Wang. Affiliation 1 should be: No.2 Office of Student Affairs, Chongqing Medical University, Chongqing, China.
