# Supplementary material for: Smoking Is Associated with an Increased Risk of Dementia: A Meta-Analysis of Prospective Cohort Studies with Investigation of Potential Effect Modifiers
Source: PLoS One. 2015 Mar 12;10(3):e0118333. doi: 10.1371/journal.pone.0118333 (PMC4357455; doi:10.1371/journal.pone.0118333)
Supplement: S1 Table — (DOC) [file pone.0118333.s003.doc]

| Table S1 Characteristics of 37 included studies regarding smoking and risk of dementia | | | | | | | | | |
| --- | --- | --- | --- | --- | --- | --- | --- | --- | --- |
| Source/study location | Female%/ loss to follow-  up rate | SS/  follow-  up^1^ | Age^2^ | Cases | Source of cohort | Smoking category | Outcome | Diagnosis criteria | Adjustment factors^4^ |
| Bowen et al [42], 2012;  USA | 58.52%; 4.17% | 808;  5y | 77.5 | 277 | Population-based | Current, former | Dementia | Unclear^3^ | Age, sex, education, APOE ε4, BMI, alcohol, hypertension, diabetes, other |
| Zhou et al  [67], 2011;  China | 42.69%; 11.68% | 2019;  5y | 72.2 | 132 | Population-based | Current, former | AD | NINCDS-  ADRDA | Age, sex, education |
| Rusanen et al [4], 2011;  USA | 56.96%;  36.20% | 21123;  30y | 58.0 | Dementia:  5376;  AD:1136;  VaD:416 | Population-based | Current, former | Dementia, AD, VaD | ICD-9-CM | Age, sex, education, BMI, alcohol, hypertension, diabetes, other |
| Ronnemaa et al [68], 2011;  Sweden | 0%;  NA | 2268^5^;  40y | 50.0 | Dementia:  349;  AD:127;  VaD:81 | Population-based | Ever | Dementia, AD, VaD | Dementia: DSM-IV; AD:NINCDS-ADRDA; VaD:ADDTC core criteria | Age, education |
| Ogunniyi et al [69], 2011;  Nigeria | 69.02%; 35.50% | 1753;  6y | 76.2 | 120 | Population-based | Ever | Dementia | ICD-10, DSM-III-R | None |
| Lin et al  [33], 2011;  USA | 43.66%;  0% | 639;  18y | 63.7 | 58 | Population-based | Current, former | Dementia | DSM-III-R | None |
| Kimm et al  [5], 2011;  Korea | 0%;  NA | 490445;  14y | 51.9 | Dementia:  3252;  AD:1851;  VaD:610 | Population-based | Current, former | Dementia, AD, VaD | Dementia: DSM-IV;  AD:ICD-10;  VaD: ICD-10 | Age, alcohol |
|  | 100%;  NA | 358060;  14y | 53.6 |  |  |  |  |  |  |
| Gao et al  [70], 2011;  USA | 71.45%;  NA | 1331;  10y | 82.2 | 207 | Population-based | Current, former | Dementia | ICD-10, DSM-III-R | None |
| Chen et al  [71], 2011;  China | NA;  5.28% | 1238;  7.5y | >65.0 | 80 | Population-based | Current, former | Dementia | GMS-  AGECAT, DSM- III | Age, sex |
| Brian et al  [35], 2011;  Australia | 0%;  1.28% | 12047;  13.4y | 72.1 | 1271 | Population-based | Current, former | Dementia | ICD-9, ICD-10 | None |
| Rusanen et al [6], 2010;  Finland | 62.65%;  27.55% | 1449;  26y | 50.6 | Dementia:  59;  AD:46 | Population-based | Current, former | Dementia, AD | Dementia: DSM-IV; AD:NINCDS-ADRDA | APOE ε4, BMI, diabetes, other |
| Scarmeas et al [72], 2009;  USA | 68.78%;  16.33% | 1880;  14y | 77.2 | 282 | Population-based | Ever | AD | NINCDS-  ADRDA | None |
| Hassing et al [29], 2009;  Sweden | 69.00%;  21.37% | 1152;  40y | 52.5 | Dementia:  312;  AD:181;  VaD:69 | Population-based | Ever | Dementia, AD, VaD | Dementia:  DSM-III-R AD:NINCDS-ADRDA; VaD: NINDS-  AIREN | None |
| Alonso et al [73], 2009;  USA | 57.47%;  NA | 1115^e^;  14y | 56.5 | 203 | Population-based | Current, former | Dementia | ICD-9 | Age, sex, education, APOE ε4, BMI, hypertension, diabetes, other |
| Kivipelto et al [74], 2008;  Finland | 61.25%;  11.39% | 1284;  27y | 50.1 | 57 | Population-based | Ever | Dementia | DSM-IV | Age, sex, education, APOE ε4, BMI, diabetes, other |
| Dahl et al  [75], 2008;  Finland | 60.50%;  49.41% | 605;  8y | 70.8 | 86 | Population-based | Ever | Dementia | DSM-IV | None |
| Beydoun et al [76], 2008;  USA | 36.53%;  22.73% | 2322;  >20y | 57.8 | 187 | Population-based | Current, former | AD | NINCDS-  ADRDA | None |
| Reitz et al  [7],2007; Netherlands | 61.46%;  0.10% | 6868;  14y | 69.5 | Dementia:  706;  AD:555;  VaD:79 | Population-based | Current, former | Dementia, AD, VaD | Dementia: DSM-III-R; AD: NINCDS- ADRDA; VaD: NINDS- AIREN | Age, sex, education, alcohol |
| Laurin et al  [36], 2007;  USA | 0%;  26.23% | 2588;  7.8y | 76.9 | 240 | Population-based | Current, former | Dementia | DSM-III-R | None |
| Aggarwal et al [8], 2006;  USA | 61.90%;  36.93% | 1064;  6.9y | 73.8 | 170 | Population-based | Current, former | AD | NINCDS- ADRDA | Age, sex, education, APOE ε4, other |
| Whitmer et al [31], 2005;  USA | 54.15%;  10.31% | 9217;  9y | 42.5 | 713 | Hospital- based | Ever | Dementia | ICD-9 | None |
| Rosengren et al [22], 2005;  Sweden | 0%;  0.35% | 7376;  24y | 51.5 | 254 | Hospital- based | Current, former | Dementia | ICD-8, ICD-9, ICD-10 | Age |
| Cherubini et al [77], 2005;  Italy | 56.00%;  10.56% | 1033;  NA | 75.5 | 58 | Population-based | Current, former | Dementia | DSM-IV | None |
| Moffat et al [34], 2004;  USA | 0%;  NA | 574;  37y | 66.3 | Dementia:  68;  AD:43 | Population-based | Ever | Dementia, AD | Dementia: DSM-III-R; AD:NINCDS-ADRDA | Age, education, BMI, diabetes, other |
| Laurin et al  [37], 2004;  USA | 0%;  25.08% | 2341;  9y | 77.4 | 235 | Population-based | Current, former | Dementia | DSM-III-R | None |
| Juan et al  [9], 2004;  China | NA;  NA | 2820^e^;  2y | 66.9 | Dementia:  121;  AD:84;  VaD:16 | Population-based | Current, former | Dementia, AD, VaD | Dementia: DSM-III-R; AD:NINCDS-ADRDA; VaD: NINDS- AIREN | Age, sex, education, alcohol, other |
| Tyas et al  [10], 2003;  USA | 0%;  13.44% | 3232;  6y | 77.7 | Dementia:  297  AD:113;  VaD:85 | Population-based | Current, former | Dementia, AD, VaD | Dementia: DSM-III-R; AD:NINCDS-ADRDA; VaD: ADDTC core criteria | Age, education, APOE ε4, alcohol, hypertension, other |
| Laurin et al  [30], 2003;  Canada | 65.80%;  NA | 163;  5y | 78.8 | 52 | Population-based | Ever | Dementia | DSM-IV | None |
| Lindsay et al [78], 2002;  Canada | 57.97%;  38.25% | 3973;  5y | 73.3 | 194 | Population-based | Ever | AD | DSM-IV | Age, sex, education |
| Tyas et al  [79], 2001;  Canada | 62.40%;  38.02% | 644;  5y | 74.0 | 36 | Population-based | Ever | AD | NINCDS-  ADRDA | None |
| Wang et al  [32], 1999;  Sweden | 81.34%;  21.69% | 343;  3y | 84.0 | Dementia:  46;  AD:34 | Population-based | Ever | Dementia, AD | DSM-III-R with minor modification | Age, sex, education |
| Merchant et al [54], 1999;  USA | 68.74%;  21.10% | 1062;  >2y | 75.4 | 142 | Population-based | Current, former | AD | NINCDS-  ADRDA | None |
| Launer et al [28], 1999; France, Denmark, UK Netherlands | NA;  20.82% | 12843;  ≧5y | >65.0 | Dementia:  400;  AD:277 | Population-based | Current, former | Dementia, AD | Dementia: DSM-III-R; AD:NINCDS-ADRDA; | Age, sex, education, other |
| Broe et al  [27], 1998;  Australia | 49.50%;  8.56% | 299;  3y | 83.4 | Dementia:  47;  AD:29 | Population-based | Current, former^6^ | Dementia, AD | Dementia: DSM-III-R; AD:NINCDS-ADRDA; | Age, sex, education |
| Yoshitake et al  [80], 1995;  Japan | 59.69%;  0.24% | 826;  7y | 73.6 | AD:42;  VaD:50 | Population-based | Ever | AD, VaD | AD:NINCDS-ADRDA; VaD: NINDS- AIREN | Age |
| Letenneur et al [81], 1994;  France | 58.25%;  NA | 3770^e^;  NA | >65.0 | 79 | Population-based | Ever, current, former | AD | NINCDS-  ADRDA | Age, sex, education, other |
| Hebert et al [82], 1992;  USA | 55.75%;  25.65% | 513;  3y | >65.0 | 76 | Population-based | Ever | AD | NINCDS-  ADRDA | Age, sex, education |

SS, sample size; APOE ε4, apolipoprotein E ε4; NA, not available; BMI, body mass index; IGT, impaired glucose tolerance; NINCDS-ADRDA, DSM-III-R, Diagnostic and Statistical Manual of Mental Disorders, third edition Revised; DSM-IV, Diagnostic and Statistical Manual of Mental Disorders, fourth edition; NINDS-AIREN, National Institute of Neurological Disorders and Stroke-Association Internationale pour la Recherche et l'Enseignement en Neurosciences; GMS-AGECAT, Geriatric Mental State-the Automated Geriatric Examination for Computer Assisted Taxonomy; ICD-8, International Classification of Diseases, Eighth Revision; ADDTC core criteria, Alzheimer’s Disease Diagnostic and Treatment Centers core criteria.

^1^ Value is expressed as maximum.

^2^ Value refers to mean age of participants at baseline.

^3^ Dementia was determined by a battery of neuropsychological measures and a standardized neurological examination.

^4^ The term “other” in the “Adjustment factors” column refers to all the confounders except age, sex, education, APOE ε4, BMI, diabetes, alcohol and hypertension.

^5^ Value refers to sample size at baseline.

^6^ The risk estimates were available just for former smoking and the risk of all-cause dementia and AD.
