# Supplementary material for: Smoking Is Associated with an Increased Risk of Dementia: A Meta-Analysis of Prospective Cohort Studies with Investigation of Potential Effect Modifiers
Source: PLoS One. 2015 Mar 12;10(3):e0118333. doi: 10.1371/journal.pone.0118333 (PMC4357455; doi:10.1371/journal.pone.0118333)
Supplement: S2 Table — (DOC) [file pone.0118333.s004.doc]

| Table S2 The results of quality assessment | | | | | | | | | | |
| --- | --- | --- | --- | --- | --- | --- | --- | --- | --- | --- |
| Source(reference) | Selection^1^ | | | | Comparability^2^ | | Outcome^3^ | | | Total Score^4^ |
|  | Representativ-eness of exposed cohort  ☆ | Selection of non-exposed cohort  ☆ | Exposure ascertainment  ☆ | No history of disease  ☆ | Comparable  on confounders  ☆☆ | | Outcome  Assessment  ☆ | Adequate follow-up  (≧10y)  ☆ | Loss to follow-up rate (≦20%)  ☆ |  |
| Bowen et al [42] |  | ☆ | ☆ | ☆ | ☆ | ☆ | ☆ |  | ☆ | 7 |
| Zhou et al [67] | ☆ | ☆ | ☆ | ☆ | ☆ | ☆ | ☆ |  | ☆ | 8 |
| Rusanen et al [4] | ☆ | ☆ | ☆ | ☆ | ☆ | ☆ | ☆ | ☆ |  | 8 |
| Ronnemaa et al [68] | ☆ | ☆ | ☆ | ☆ | ☆ |  | ☆ | ☆ |  | 7 |
| Ogunniyi et al [69] | ☆ | ☆ | ☆ | ☆ |  |  | ☆ |  |  | 5 |
| Lin et al [33] |  | ☆ |  | ☆ |  |  | ☆ | ☆ | ☆ | 5 |
| Kimm et al [5] | ☆ | ☆ | ☆ | ☆ | ☆ |  | ☆ | ☆ |  | 7 |
| Gao et al [70] | ☆ | ☆ | ☆ | ☆ |  |  | ☆ | ☆ |  | 6 |
| Chen et al [71] | ☆ | ☆ | ☆ | ☆ | ☆ | ☆ | ☆ |  | ☆ | 8 |
| Brian et al [35] | ☆ | ☆ | ☆ | ☆ |  |  | ☆ | ☆ | ☆ | 7 |
| Rusanen et al [6] | ☆ | ☆ | ☆ | ☆ | ☆ |  | ☆ | ☆ |  | 7 |
| Scarmeas et al [72] | ☆ | ☆ | ☆ | ☆ |  |  | ☆ | ☆ | ☆ | 7 |
| Hassing et al [29] |  | ☆ | ☆ | ☆ |  |  | ☆ | ☆ |  | 5 |
| Alonso et al [73] | ☆ | ☆ |  | ☆ | ☆ | ☆ | ☆ | ☆ |  | 7 |
| Kivipelto et al [74] | ☆ | ☆ |  | ☆ | ☆ | ☆ | ☆ | ☆ | ☆ | 8 |
| Dahl et al [75] |  | ☆ | ☆ | ☆ |  |  | ☆ |  |  | 4 |
| Beydoun et al [76] | ☆ | ☆ |  | ☆ |  |  | ☆ | ☆ |  | 5 |
| Reitz et al [7] | ☆ | ☆ | ☆ | ☆ | ☆ | ☆ | ☆ | ☆ | ☆ | 9 |
| Laurin et al [36] | ☆ | ☆ | ☆ | ☆ |  |  | ☆ |  |  | 5 |
| Aggarwal et al [8] | ☆ | ☆ | ☆ | ☆ | ☆ | ☆ | ☆ |  |  | 7 |
| Whitmer et al [31] |  | ☆ | ☆ | ☆ |  |  | ☆ |  | ☆ | 5 |
| Rosengren et al [22] |  | ☆ |  | ☆ | ☆ |  | ☆ | ☆ | ☆ | 6 |
| Cherubini et al [77] | ☆ | ☆ | ☆ | ☆ |  |  | ☆ |  | ☆ | 6 |
| Moffat et al [34] |  | ☆ |  | ☆ | ☆ |  | ☆ | ☆ |  | 5 |
| Laurin et al [37] | ☆ | ☆ | ☆ | ☆ |  |  | ☆ |  |  | 5 |
| Juan et al [9] | ☆ | ☆ | ☆ | ☆ | ☆ | ☆ | ☆ |  |  | 7 |
| Tyas et al [10] | ☆ | ☆ | ☆ | ☆ | ☆ |  | ☆ |  | ☆ | 7 |
| Laurin et al [30] |  | ☆ | ☆ | ☆ |  |  | ☆ |  |  | 4 |
| Lindsay et al [78] | ☆ | ☆ | ☆ | ☆ | ☆ | ☆ | ☆ |  |  | 7 |
| Tyas et al [79] |  | ☆ | ☆ | ☆ |  |  | ☆ |  |  | 4 |
| Wang et al [32] |  | ☆ |  | ☆ | ☆ | ☆ | ☆ |  |  | 5 |
| Merchant et al [54] | ☆ | ☆ | ☆ | ☆ |  |  | ☆ |  |  | 5 |
| Launer et al [28] | ☆ | ☆ | ☆ | ☆ | ☆ | ☆ | ☆ |  |  | 7 |
| Broe et al [27] |  | ☆ |  | ☆ | ☆ | ☆ | ☆ |  | ☆ | 6 |
| Yoshitake et al [80] |  | ☆ | ☆ | ☆ | ☆ |  | ☆ |  | ☆ | 6 |
| Letenneur et al [81] | ☆ | ☆ | ☆ | ☆ | ☆ | ☆ | ☆ |  |  | 7 |
| Hebert et al [82] |  | ☆ | ☆ | ☆ | ☆ | ☆ | ☆ |  |  | 6 |

^1^ ”Selection” part includes representativeness of cases, selection of controls, exposure ascertainment, and no history of disease.

^2^ ”Comparability” part includes comparable on confounders.

^3^ ”Outcome” part includes outcome assessment, adequate follow-up, and loss to follow-up rate.

^4^ The total score is equal to the total number of stars.
